# Dried chicory root improves bowel function, benefits intestinal microbial trophic chains and increases faecal and circulating short chain fatty acids in subjects at risk for type 2 diabetes

**DOI:** 10.1017/gmb.2022.4

**Published:** 2022-04-28

**Authors:** Marie-Luise Puhlmann, Roosa Jokela, Katja Catharina Wilhelmina van Dongen, Thi Phuong Nam Bui, Roland Willem Jan van Hangelbroek, Hauke Smidt, Willem Meindert de Vos, Edith Johanna Maria Feskens

**Affiliations:** 1 Laboratory of Microbiology, Wageningen University & Research, Wageningen, The Netherlands; 2 Division of Human Nutrition and Health, Wageningen University & Research, Wageningen, The Netherlands; 3 Human Microbiome Research Program, Faculty of Medicine, University of Helsinki, Helsinki, Finland; 4 Division of Toxicology, Wageningen University & Research, Wageningen, The Netherlands; 5 Caelus Health, Amsterdam, The Netherlands; 6 Department of Data Science, Euretos BV, Utrecht, The Netherlands

**Keywords:** Chicory root, intrinsic fibre, gut microbiota, short-chain fatty acids, type 2 diabetes, *Blautia*

## Abstract

We investigated the impact of dried chicory root in a randomised, placebo-controlled trial with 55 subjects at risk for type 2 diabetes on bowel function, gut microbiota and its products, and glucose homeostasis. The treatment increased stool softness (+1.1 ± 0.3 units; *p =* 0.034) and frequency (+0.6 ± 0.2 defecations/day; *p* < 0.001), strongly modulated gut microbiota composition (7 % variation; *p =* 0.001), and dramatically increased relative levels (3-4-fold) of *Anaerostipes* and *Bifidobacterium* spp., in a dose-dependent, reversible manner. A synthetic community, including selected members of these genera and a *Bacteroides* strain, generated a butyrogenic trophic chain from the product. Faecal acetate, propionate and butyrate increased by 25.8 % (+13.0 ± 6.3 mmol/kg; *p* = 0.023) as did their fasting circulating levels by 15.7 % (+7.7 ± 3.9 μM; *p =* 0.057). In the treatment group the glycaemic coefficient of variation decreased from 21.3 ± 0.94 to 18.3 ± 0.84 % (*p =* 0.004), whereas fasting glucose and HOMA-ir decreased in subjects with low baseline *Blautia* levels (−0.3 ± 0.1 mmol/L fasting glucose; *p =* 0.0187; −0.14 ± 0.1 HOMA-ir; *p =* 0.045). Dried chicory root intake rapidly and reversibly affects bowel function, benefits butyrogenic trophic chains, and promotes glycaemic control.

## Introduction

Fibre intake has increasingly been recognised as a major factor in the maintenance of human health and contributes to lowering risk for metabolic diseases like type 2 diabetes (T2D) (Reynolds et al., 2019, 2020). However, only a small percentage of the general population consumes the recommended fibre amount, a problem known as the fibre gap (Jones, 2014). Since most fibres reach the colon in an undigested form, they impact the composition and activity of the gut microbiota. This is highly relevant since specific microbiota signatures are associated with various stages of T2D (De Vos and Nieuwdorp, 2013) mirroring increased insulin resistance in prediabetes and T2D by shifts towards reduced relative abundance of butyrate-producing bacteria (Wu et al., 2020). A study revisiting several dozens of T2D cohorts revealed a decrease in relative levels of butyrate-producing bacteria and an increase of *Blautia, Ruminococcus* and *Fusobacterium* spp. (Gurung et al., 2020). Moreover, intake of a complex fibre mixture in Chinese T2D patients improved glucose homeostasis concomitantly with increased levels of butyrate-producing bacteria (Zhao et al., 2018).

The gut microbiota uses fibres as carbon and energy source, resulting in the production of short-chain fatty acids (SCFA), such as acetate, propionate and butyrate. These SCFA not only exert local effects by serving as fuel for colonocytes and interacting with G-protein-coupled receptors (GPRs) but might also enter the systemic circulation, directly interacting with peripheral tissue function and thereby affecting metabolism (Canfora et al., 2015). Prediabetic subjects receiving colonic infusions of SCFA mixtures at concentrations and ratios reached after fibre intake improved metabolic health parameters particularly when SCFA were administered in the distal colon (Canfora et al., 2017; van der Beek et al., 2016). Such a distal shift in fermentation might also benefit treatment of functional bowel diseases (So et al., 2021). Consequently, modulating the gut microbiota towards increased SCFA production might offer new therapeutic avenues for metabolic diseases (Canfora et al., 2015; Fan and Pedersen, 2020; Gurung et al., 2020).

Especially well-studied fibres are inulin-type fructans (ITF) (Swanson et al., 2020), including isolated and purified native inulin from chicory roots. Consumption of ITF is known to stimulate faecal levels of *Bifidobacterium* spp. (Le Bastard et al., 2019) as well as to contribute to maintaining bowel function, which has resulted in a validated health claim (EFSA, 2015). However, ITF are reported to have a rather limited effect on modulating gut microbiota (Dewulf et al., 2013; Kiewiet et al., 2021) and a moderate to no effect on faecal and circulating SCFA and insulin resistance markers (Chambers et al., 2015, 2019). A recent study reviewing interventions with ITF, other single fibres and whole grain foods concluded that total faecal SCFA were not impacted (So et al., 2018). Of note, it has been suggested that consumption of plant and fibre-rich foods would increase fibre-degrading and SCFA-producing gut bacteria (Koponen et al., 2021). While studying the effect of single or purified dietary components is the conventional way to obtain mechanistic insights, this reductionist approach does not take into account that dietary fibres do not exist in isolation in minimally and unprocessed products. They are either part of the plant cell wall (pectin, hemicellulose and cellulose) or encapsulated by it (storage carbohydrates such as inulin), hence termed intrinsic fibres (Augustin et al., 2020)^.^ This intrinsic structure of fibres is likely to result in a gradual release of fermentable carbohydrates and hence a rather distal intestinal location where SCFA are produced and absorbed (Hansen and Sams, 2018; So et al., 2021). We therefore hypothesised that intrinsic fibres substantially increase faecal SCFA levels in the distal colon and possibly affect health differently from purified fibres (Dagbasi et al., 2020; Grundy et al., 2016; Hansen and Sams, 2018).

Previously, we showed that chicory roots have a long history of consumption and contain the highest dry fibre content of edible vegetables, nuts, seeds and fruits (Puhlmann and de Vos, 2020). In the present study, we aimed to explore the health effects of dried chicory roots that contain approximately 85 % fibre, consisting of native inulin enclosed in plant cell wall fibres (pectin, cellulose and hemicellulose). We assessed the effect of daily dried chicory root intake on bowel function, gut microbiota composition, faecal and circulating SCFA levels, as well as glucose homeostasis markers in subjects at risk of T2D in a randomised, parallel, placebo-controlled, and investigator-blinded trial.

## Methods

### Participants

Participants were locally recruited in the surroundings of Wageningen University & Research in Wageningen, The Netherlands, between May and September 2018. Participants were eligible if they were between the age of 40–75 years and had either a fasting blood glucose level between 5.0 and 5.6 mmol/L with a high risk to develop type 2 diabetes (T2D) later in life as assessed by a diabetes risk score ≥9 (Alssema et al., 2008; Lindström and Tuomilehto, 2003), or a fasting blood glucose level between 5.6 and 6.9 mmol/L (classified as prediabetes by the American Diabetes Association, 2016). Randomisation was performed by a researcher not involved in the study. Participants were randomly assigned by a third researcher to either the treatment or the placebo group within strata based on sex and fasting glucose level inclusion criteria. History of medical or surgical events that may significantly affect the study outcome, medical drug use for T2D or chronic use of antacids resulted in exclusion from the study. Other exclusion criteria included the use of antibiotics during the three months prior to screening and consumption of pre- or probiotics or any fibre supplement during the last month prior to screening. Participants were also excluded if they reported any unexplained weight loss or weight gain of more than 5 kg in the 1 month prior to the screening, or if they followed a slimming or medically prescribed diet, or a vegan or macrobiotic lifestyle. Finally, any known sensitivity to medical skin adhesives also led to exclusion from participation. In total 156 men and women were screened, whereof 63 participants were eligible and 60 were included in the study.

### Study design

**Figure 1:**
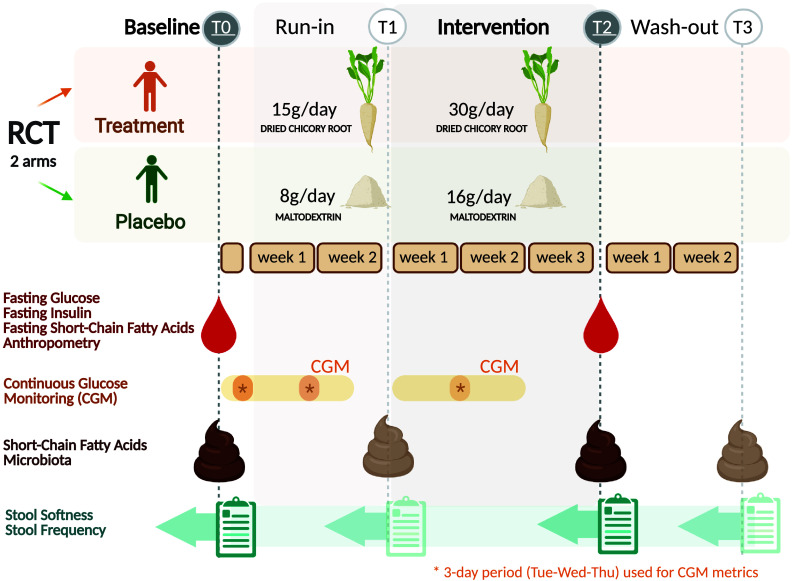
Trial design of the randomised placebo-controlled parallel trial comparing dried chicory root intake with maltodextrin placebo. Asterisks indicate 3-day period (same weekdays) used for the calculation of continuous glucose monitoring metrics for baseline (T0), run-in (T1) and intervention period (T2). Created with BioRender.com.

The study was a two-arm randomised, placebo-controlled, investigator-blinded, parallel trial. The study consisted of three study periods, a run-in of 2 weeks, an intervention period of 3 weeks and a wash-out of 2 weeks (Figure 1). A detailed description of the study design is given in the Supplemental Methods. The Medical Ethical Review Committee Wageningen University (METC-WU nr. 17/25) approved this study and was registered at the ISRCTN registry (ISRCTN39985847). The authors assert that all procedures contributing to this work comply with the ethical standards of the relevant national and institutional committees on human experimentation and with the Helsinki Declaration of 1975, as revised in 2008.

Primary outcomes were defined as changes in the static glycaemic markers HOMA-ir, fasting insulin and fasting glucose after the study period (30 g/day treatment intake; T2) against baseline (T0). Secondary outcomes were differences in changes over time between intervention groups in bowel function assessed by stool softness (Bristol Stool Scale, BSS) (Lewis and Heaton, 1997) and stool frequency (defecations per day), gut microbiota composition, faecal and fasting circulating SCFA, and glycaemic variability metrics captured by continuous glucose monitoring (CGM) after the study period (T2) and in addition after the run-in (T1) and the wash-out period (T3).

### Intervention products

The treatment product consisted of WholeFiber (provided by WholeFiber BV, The Netherlands), an intrinsic multifibre product made from chicory root that are washed, cut and dried. The dried chicory root product has a dry weight of 93 % (w/w), including 85 % (w/w) fibre, whereof 70 % (w/w) is native inulin, 10 % (w/w) pectin and 5 % (w/w) hemicellulose and cellulose. This treatment product was taken at a daily dose of 30 g for 3 weeks in the treatment group, while the placebo group consumed a daily dose of an iso-caloric amount of 16 g maltodextrin (Body&Fit, The Netherlands). During the run-in period, both groups received half the amount, 15 g of the treatment product and 8 g of the placebo, respectively.

### Microbiota composition, *in* *vitro* experiments, biochemical analyses and bowel function diaries

Details about the analysis of gut microbiota composition using 16S rRNA amplicon sequencing, synthetic co- and tri-culture experiments, assessment of bowel function (stool softness and frequency), faecal and fasting circulating SCFA, static (fasting insulin, fasting glucose and HOMA-ir), as well as dynamic (CGM) glycaemic markers can be found in the Supplemental Methods and Supplemental Table S1. Gut microbiota data has been submitted to the European Nucleotide Archive (ENA) under accession number PRJEB47230.

### Statistical analysis and subgroup segmentation

Data is presented as mean ± SEM if not indicated differently. For the purpose of comparison with other studies, changes were also expressed as percentage change (absolute change divided by baseline value). GraphPad Prism 9.1 and *R* version 4.0.3 were used to generate corresponding graphics and for statistical analysis applying *α* = 0.05. Gut microbiota data was analysed using the R package mare (Korpela, 2016). Differences in changes in faecal SCFA, bowel function and CGM metrices were analysed with repeated-measures analyses using the lme4 R package (Bates et al., 2015) or the rstatix R package (Kassambara, 2021). Post-intervention differences in plasma biomarkers and others were assessed by analysis of covariance. Sample size calculation, further details on the statistical analyses, on responder analyses and on subgroup segmentation are given in the Supplemental Methods and Supplemental Figure S1.

## Results

### Baseline characteristics

In total, 58 participants completed the study as two participants dropped out for personal reasons and another three were excluded due to lifestyle changes, protocol violation or missing samples (Supplemental Figure S2). Product intake compliance, assessed by returned empty and leftover sachets, was excellent (96.3 per cent). At baseline, we observed that the placebo and treatment group were well randomised, but the placebo group had slightly softer stools (Table 1). While fasting plasma glucose levels were elevated (f-glu_all_ = 6.0 ± 0.6 mmol/L) according to prediabetes criteria (American Diabetes Association, 2016), overall insulin resistance as assessed by HOMA-ir was relatively low in the study population (HOMA-ir_all_ = 1.31 ± 0.61).

**Table 1.** Baseline characteristics of all subjects included in the data analysis.

	Treatment (*n* = 28)	Placebo (*n* = 27)	*p*-value
Sex			
Female, *n*	14	14	
Male, *n*	14	13	
Age, years, median (IQR)	69 (52−75)	69 (48−75)	0.76
Fibre intake (g/day), mean (±SD)	23.6 (7.6)	22.0 (8.2)	0.44
BMI (kg/m^2^), mean (±SD)	28.6 (3.7)	28.3 (3.1)	0.75
Weight (kg), mean (±SD)	85.0 (17.7)	84.7 (14.6)	0.94
Waist (cm), mean (±SD)	102.7 (12.2)	103.3 (10.0)	0.86
Glucose (mmol/L), mean (±SD)	6.1 (0.6)	5.9 (0.6)	0.20
Insulin (μU/mL), mean (±SD)	8.05 (3.86)	8.57 (3.99)	0.63
HOMA-ir, median (IQR)	1.1 (0.4−2.7)	1.2 (0.4−3.6)	0.52
CV (%), mean (±SD)	21.4 (4.81)	20.6 (5.57)	0.55
Stool Frequency (x/day), median (IQR)	1.0 (0.9−3.0)	1.0 (0.3−2.5)	0.31
Stool Softness (BSS), mean (±SD)	3.3 (1.3)	4.1 (1.2)	0.02

Abbreviations: BSS, Bristol stool score; CV, coefficient of variation assessed by continuous glucose measurement.

### Bowel function

First, we wanted to understand how dried chicory root would affect gut function by assessing bowel habits recorded as stool softness and stool frequency. We observed that the treatment had a pronounced effect over time on stool softness (repeated-measures ANOVA interaction: *F*(3,159) = 2.952, *p =* 0.034) and stool frequency (Friedman test: *χ* ^2^(3) = 22.3, *p* < 0.001). Stool softness increased by +1.1 ± 0.3 units (BSS; *p =* 0.034) from 3.3 ± 0.3 to 4.4 ± 0.2 after 3 weeks of 30 g/day treatment (T2), a change that already started after 15 g/day (T1: + 0.6 ± 0.3 BSS, *p =* 0.072). Of note, stool frequency increased with +0.6 ± 0.2 from 1.3 ± 0.1 to 1.9 ± 0.1 defecations per day after 30 g/day treatment (T2; *p =* 0.004), an increase that also started at 15 g/day (T1: +0.2 ± 0.1×/day, *p =* 0.038). In contrast, in the placebo stool softness remained constant with 4.1 ± 0.2 at baseline, 4.3 ± 0.2 after run-in (T1; *p =* 0.69) and 4.2 ± 0.2 after study period (T2; *p =* 0.69). Similar, stool frequency remained constant with 1.2 ± 0.1 defecations/day at baseline and 1.3 ± 0.2 after run-in (T1; *p =* 0.347) and 1.3 ± 0.1 study period (T2; *p =* 0.582). For both groups, stool softness and frequency returned to baseline levels after 2 weeks wash-out (T3 treatment: BSS = 3.5 ± 0.2, stool frequency = 1.2 ± 0.1 and placebo: BSS = 4.1 ± 0.2, stool frequency = 1.3 ± 0.1). These increasing changes in bowel function over time and related fibre dosages indicated a substantial modulatory potential of dried chicory roots on the colonic environment.

### Gut microbiota composition

**Figure 2:**
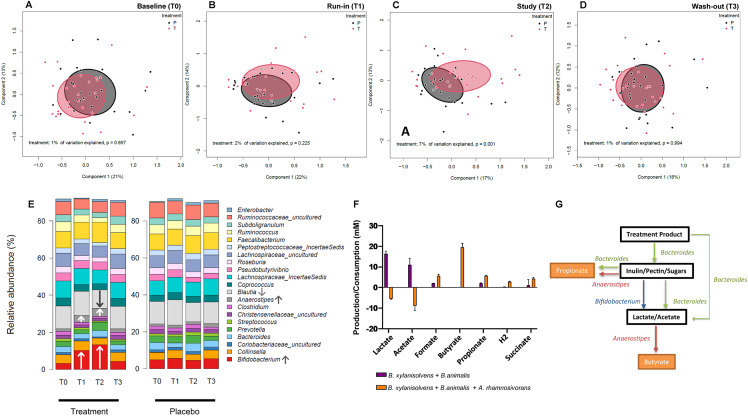
(A–D) Effect of dried chicory root consumption on overall gut microbiota composition [β-diversity, assessed by principal coordinates analysis (PCoA) with Bray–Curtis dissimilarity] at baseline (T0) after 2 weeks of 15 g/day treatment or iso-caloric placebo (T2), 3 weeks of 30 g/day treatment or iso-caloric placebo (T2) and after 2 weeks of wash-out (T3). No changes in *α*-diversity measures were observed, which reflects the within-person variation in microbiota composition by measuring the number (richness) of different bacterial taxa and their distribution (evenness) within a sample. (E) Gut microbiota composition at baseline (T0), 15 g/day treatment or iso-caloric placebo (T1), 30 g/day treatment or iso-caloric placebo (T2) and washout (T3). We observed a significant increase (↑) of *Bifidobacterium* spp. and *Anaerostipes* spp. at T1 and T2 in the treatment group and a significant decrease (↓) of *Blautia* spp. at T2. None of the taxa in the placebo group changed significantly between any of the timepoints. (F) Synthetic co- and tri-culture experiments. Metabolite production and consumption in a tri-culture containing *Bacteroides xylanisolvens* (*B. xylanisolvens*), *Bifidobacterium animalis* subsp. *lactis* BLC1 (*B. animalis* BLC1) and *Anaerostipes rhamnosivorans* 1y2T (*A. rhamnosivorans*) incubated with the treatment product consisting of dried chicory roots (5 g/L in YCFA) (Duncan et al., 2009). Mean values are shown with standard deviation after 7 days incubation at 37°C but 80 % of the conversion was already completed after 3 days of incubation (not shown). (G) Postulated microbial and metabolic interaction network involved in the colonic fermentation of the dried chicory roots (predominantly consisting of inulin, pectin and hemicellulose and cellulose). Proposed conversion by *Bacteroides* spp. is presented in green, by *Bifidobacterium* spp. in blue and by *Anaerostipes* spp. in red. The treatment product, dried chicory roots, consists of plant cells, which are envisaged to be degraded and liberate the intermediate products inulin, pectin and small sugars (like fructo-oligosaccharides) that are further converted into the end products (highlighted in orange) including propionate or butyrate via the intermediate products lactate and acetate.

To understand the effect of dried chicory root intake on the gut microbes, we then assessed the overall faecal microbiota composition as well as changes in the relative abundance of individual genera. We observed a strong modulatory effect of the treatment product on overall gut microbiota composition assessed by Bray–Curtis β-diversity (Figure 2A–D), which in this context reflects the between-person differences in gut microbiota composition. Already after 2 weeks of 15 g/day treatment (T1; Figure 2B), overall gut microbiota composition of the treatment and placebo shifted apart, finally explaining 7 % (Permutational multivariate analysis of variance (PERMANOVA), *p =* 0.001) of the observed variation in microbial composition after 3 weeks of 30 g/day treatment (T2; Figure 2C). Interestingly, this effect was fully reversible as overall gut microbiota composition returned to baseline once subjects had stopped for 2 weeks with the treatment (wash-out; T3, Figure 2D). The overall compositional changes by the treatment were mirrored in the change of several taxa at genus level (Figure 2E). We observed the most pronounced changes in the stimulation of relative levels of *Anaerostipes* and *Bifidobacterium* spp. (Figure 2E and Supplemental Figure S3). Of these genera, the most abundant species were identified as *A. hadrus* and *A. butyraticus* as well as *B. longum* and an unclassified *Bifidobacterium* species. After 2 weeks of 15 g/day treatment (T1) the relative abundance of *Anaerostipes* spp. increased already by 2.82-fold (*q* < 0.001, Supplemental Table S2) reaching levels twice as high as placebo (T1: treatment = 3.7 % vs. placebo *=* 1.6 %, *q* < 0.001; Supplemental Table S3). After 30 g/day treatment *Anaerostipes* spp. relative abundance increased 3.24-fold (T2, *q* < 0.001; Supplemental Table S2) to levels three times higher than placebo (treatment = 4.3 % vs. placebo *=* 1.4 %, *q* < 0.001; Supplemental Table S3). In addition, *Bifidobacterium* spp. relative abundance increased 3.17-fold after 15 g/day treatment (T1, *q* < 0.001) and 4.09-fold after 30 g/day treatment (T2, *q* < 0.001; Supplemental Table S2) to a relative level of 13.0 %, which was also three times higher than placebo (T2: placebo *=* 4.5 %, *q* = 0.002; Supplemental Table S3). We also observed several taxa to decrease in relative abundance after treatment intake (Figure 2E and Supplemental Table S2), notably *Blautia* spp. of which the most abundant human species were identified as *B. hominis*, *B. luti*, and *B. obeum.* None of these changes were observed in the placebo group after run-in or study (*q* > 0.05, Supplemental Table S4) and no taxa differed between the groups after the washout (T3; Supplemental Table S3). In summary, the modulation of the overall gut microbiota composition and concomitant changes in relative abundance of specific taxa were dose-dependent and reversible.

### Trophic chain *in* *vitro* experiment

To demonstrate that the observed changes in relative faecal abundances of both *Bifidobacterium* and *Anaerostipes* spp. after chicory root intake were associated with a trophic chain involving these two functional bacterial groups, we performed *in vitro* experiments with synthetic co- and tri-cultures as a proof-of-concept (Figure 2F). For this purpose, we selected strains with the canonical functionality to convert inulin or ITF into lactate and acetate (*Bifidobacterium* spp.) and the capacity to convert these further into butyrate (*Anaerostipes* spp.). We incubated the treatment product at a concentration of 5 g/L with the well-studied *B. animalis* BLC1 (Bottacini et al., 2011) and *A. rhamnosivorans* 1y2T (Bui et al., 2014) and found a modest but reproducible increase of butyrate (1.19 ± 0.39 mM). Hence, we anticipated the need for the degradation of the plant cell wall fibres present in the treatment product, which is a known metabolic feature of the abundant gut bacteria *Bacteroides* and *Prevotella* spp. (Martens et al., 2011). Therefore, we also included *Bacteroides xylanisolvens* HMP, a known pectin degrader (Despres et al., 2016) in a co-culture experiment with only *B. animalis* BLC1 and in a tri-culture experiment with also *A. rhamnosivorans* 1y2T. Only when using this latter tri-culture, we observed the production of high amounts of butyrate (19.5 ± 2.0 mM; Figure 2F), representing over half of the amount that dried chicory roots potentially may provide. Hence, this proof-of-concept experiment showed that assisted by a pectin-degrader, such as the selected *Bacteroides* strain, a butyrogenic trophic chain was formed from the dried chicory root involving representative members of *Bifidobacterium* and *Anaerostipes* spp.

### Faecal SCFA

We then assessed the impact of dried chicory root on faecal SCFA levels as a measure of gut microbiota activity. Total faecal SCFA levels increased by 13.02 ± 02 mmol/kg after 30 g/day treatment for 3 weeks, a relative increase of +25.7 % which was reflected in all three SCFA (T2; Table 2). Using linear mixed modelling we confirmed that faecal SCFA levels pronouncedly changed from placebo (*p =* 0.023, interaction term intervention*period) during 30 g/day treatment (T2, Table 2; estimated model fixed effects are provided in Supplemental Table S6). The increase in acetate levels (*p =* 0.022) was highest with a relative change of +28.3 % followed by increased propionate levels by +22.3 % and butyrate levels by +19.6 % (T2: *p =* 0.065 and *p =* 0.052, respectively; Table 2). The increase in faecal SCFA levels started already after 15 g/day treatment (T1; Table 2). The mean levels of faecal butyrate in the treatment group increased most at T1 by +25.8 % compared to 19.6 % at T2 (Table 2). However, the proportion of subjects with an increase in faecal butyrate levels (minimal observed increase > 1 mM) was actually highest at T2 (18 out of 28) and differed from that in the placebo (9 out of 27; Fisher’s exact: *p =* 0.015, Supplemental Figure S4). Overall, faecal SCFA levels in the placebo slightly decreased (Table 2). Once treatment intake stopped, faecal SCFA levels returned to baseline (T3, Table 2). Hence, dried chicory root also modulated gut microbiota activity and increased faecal SCFA levels in a reversible way.

**Table 2.** Levels and changes of faecal short-chain fatty acid levels at baseline (T0), after 2 weeks of 15 g/day treatment or 8 g/day iso-caloric placebo intake (T1), after 3 weeks of 30 g/day treatment or 16 g/day iso-caloric placebo intake (T2), and after 2 weeks of wash-out (T3).^a^

	Treatment				Placebo				*p*-value Intervention × Period		
SCFA (mmol/kg)	T0	T1	T2	T3	T0	T1	T2	T3	T1 vs. T0	T2 vs. T0	T3 vs. T0
Total SCFA	50.65 (4.71)	60.21 (4.96)	63.67 (5.12)	48.91 (6.77)	56.50 (5.95)	59.62 (6.64)	50.00 (5.48)	51.49 (5.53)	0.415	0.023	0.690
Δ Total SCFA		9.56 (5.74)	13.02 (6.26)	1.76 (6.32)		2.75 (4.61)	−6.51 (6.19)	−4.75 (5.77)			
Acetate	32.67 (3.27)	38.34 (3.33)	41.92 (3.40)	31.42 (4.42)	37.49 (4.10)	39.90 (4.88)	33.05 (3.71)	33.99 (3.67)	0.544	0.022	0.637
Δ Acetate		5.67 (4.03)	9.25 (4.43)	1.51 (4.37)		2.16 (3.29)	−4.44 (4.35)	−3.68 (3.91)			
Propionate	9.07 (0.82)	10.66 (0.89)	11.09 (1.04)	9.13 (1.49)	9.28 (0.81)	10.11 (0.87)	8.83 (0.79)	9.10 (0.82)	0.553	0.065	0.955
Δ Propionate		1.60 (0.81)	2.02 (0.90)	0.15 (1.08)		0.85 (0.75)	−0.45 (0.90)	−0.01 (0.96)			
Butyrate	8.91 (1.02)	11.21 (1.17)	10.66 (1.08)	8.36 (1.16)	9.74 (1.29)	9.62 (1.21)	8.12 (1.13)	8.40 (1.27)	0.140	0.052	0.717
Δ Butyrate		2.30 (1.25)	1.75 (1.18)	0.09 (1.17)		−0.26 (1.12)	−1.62 (1.21)	−1.06 (1.22)			

Abbreviation: SCFA, short-chain fatty acids.

a Data is presented as mean (SEM). Analysis was done by linear mixed model that assessed whether the change (∆) in SCFA over intervention periods by the treatment was different from placebo (represented by the interaction term: intervention × period). All estimated model fixed effects are provided in Supplemental Table S6. Analysis of post-intervention differences between groups at T2 only are provided in Supplemental Table S7.

### Fasting circulating SCFA

To determine the effect of the increased faecal SCFA on the systemic availability of these metabolites, we also measured fasting circulating SCFA at the end of the study period with 30g/day treatment intake. We observed an increase after 30 g/day treatment in total fasting circulating SCFA of 7.70 ± 3.88 μM (T2; *p =* 0.057), which represents a relative change over baseline of +15.7 % and was a result of both increased fasting acetate (+16.4 %; *p =* 0.057) and propionate levels (11.1%; *p =* 0.431, Table 3). The placebo remained largely unchanged, with total fasting circulating SCFA slightly decreasing (−4.5 %; *p =* 0.591, Table 3), while the changes did not differ between groups (Table 3). Fasting butyrate levels decreased in the placebo group about five times more than in the treatment group, where the levels remained virtually unchanged (Table 3). Comparing adjusted post-intervention levels we found an increase in total SCFA by the treatment against placebo of +8.56 ± 4.97 μM (*p =* 0.091), for acetate of +7.47 ± 4.85 μM (*p =* 0.129), for propionate of +0.85 ± 0.76 μM (*p =* 0.266) and for butyrate of +0.06 ± 0.08 μM (*p =* 0.451; see also Supplemental Table S7). Hence, the treatment with dried chicory root also resulted in systemic effects.

**Table 3.** Fasting circulating SCFA levels at baseline (T0), after 3 weeks of 30 g/day treatment or 16 g/day iso-caloric placebo intake (T2) and change (∆) over baseline.^a^

	Treatment				Placebo				
Circulating SCFA (μM)	T0	T2	Δ_T_	*p*-value	T0	T2	Δ_P_	*p*-value	*p*-value Δ_T−_Δ_P_
Fasting total SCFA	49.10 (3.60)	56.80 (4.02)	7.70 (3.88)	0.057	51.86 (4.40)	49.53 (3.85)	−2.33 (4.29)	0.591	0.088
Fasting acetate	43.93 (3.50)	51.13 (3.92)	7.20 (3.62)	0.057	45.89 (4.43)	44.60 (3.85)	−1.29 (4.33)	0.768	0.137
Fasting propionate	4.98 (0.65)	5.53 (0.57)	0.55 (0.69)	0.431	5.65 (0.54)	4.85 (0.53)	−0.80 (0.68)	0.251	0.170
Fasting butyrate^b^	0.19 (0.09)	0.15 (0.06)	−0.04 (0.10)	0.946	0.31 (0.12)	0.08 (0.06)	−0.23 (0.14)	0.092	0.094

Abbreviation: SCFA, short-chain fatty acids.

a Data is presented as mean (SEM).

b Butyrate levels were analysed using nonparametric testing.

### Static and dynamic markers of glucose homoeostasis

Finally, we wanted to understand whether metabolic markers of glucose homeostasis were affected by the intake of dried chicory root. First, we assessed the static markers HOMA-ir, fasting insulin and fasting glucose at the end of the 30 g/day treatment compared to baseline. HOMA-ir decreased from 1.28 ± 0.12 to 1.24 ± 0.09 after 30 g/day treatment (*p =* 0.566), a relative decrease over baseline by −3.1 %. Similarly, insulin decrease slightly by −2.4 % (−0.19 ± 0.50 μU/mL, *p =* 0.710) and fasting glucose by −2.1 % (−0.13 ± 0.08 mmol/L, *p =* 0.637). Yet, none of these changes differed from those in placebo (Supplemental Table S8). Comparing adjusted post-intervention levels between groups, we found a decrease in HOMA-ir against placebo of −0.05 ± 0.09 (*p =* 0.570), which was mirrored in fasting insulin (−0.32 ± 0.6 μU/mL, *p =* 0.597) but absent in fasting glucose (−0.02 ± 0.12 mmol/L, *p =* 0.877; Supplemental Table S7).

**Figure 3:**
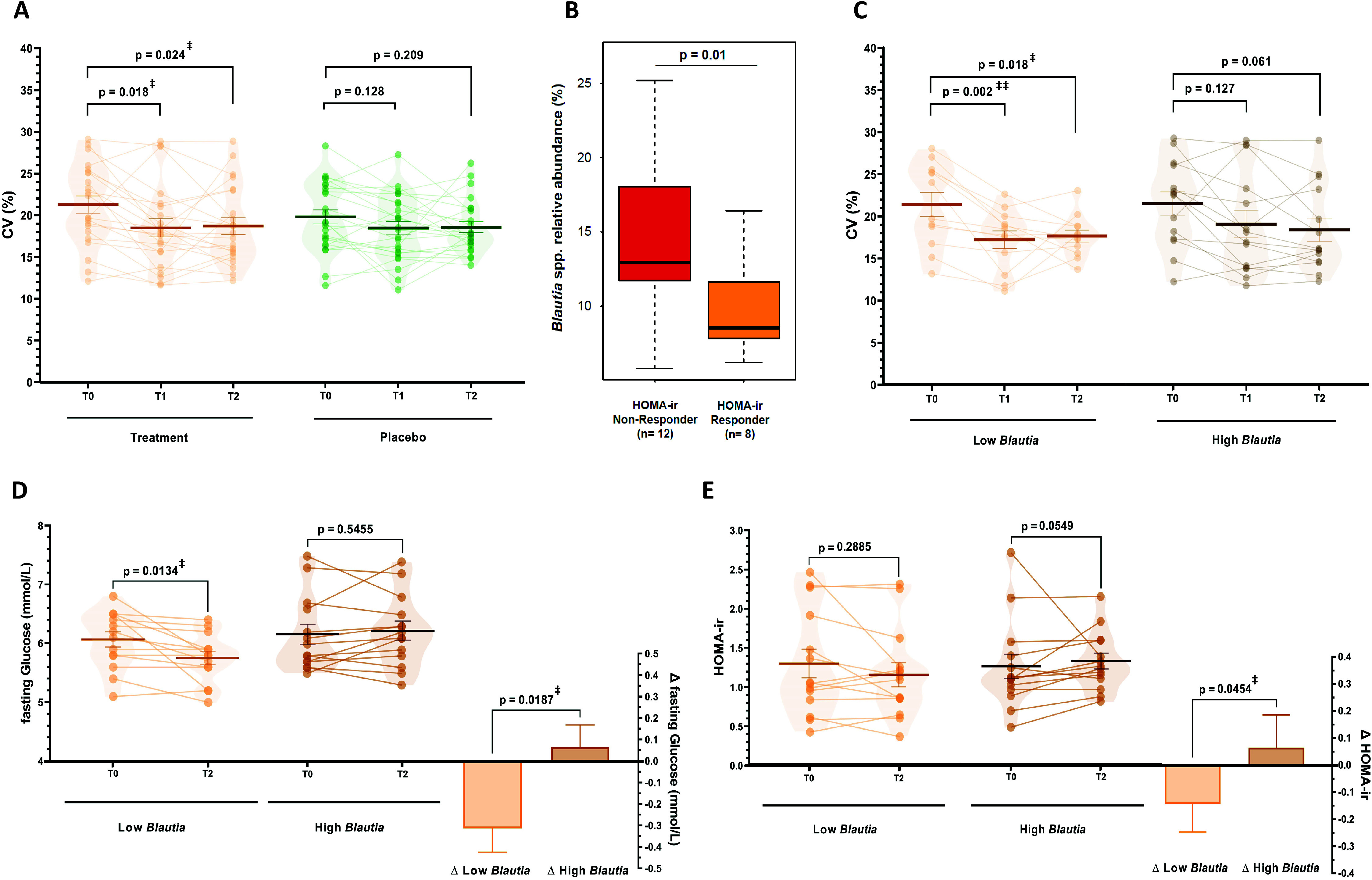
(A). Coefficient of variation (CV%) as a measure of glycaemic control assessed using continuous glucose measurement on three same consecutive weekdays during baseline (T0), the run-in period (T1) with 15 g/day treatment or 8 g/day iso-caloric placebo, and during the study period (T2) with 30 g/day treatment or 16 g/day isocaloric placebo (repeated-measures ANOVA with main effect of period *p* = 0.001, post-hoc tests with FDR-adjustment). Data is presented after sensitivity analysis excluding datasets with more than 20 % missing data and an extreme outlier (3 × IQR) for treatment (*n* = 22) and placebo group (*n* = 24). No difference between groups in baseline CV was observed before (*p* = 0.55) and after sensitivity analysis (*p* = 0.25). (B) Difference in relative abundance of *Blautia* spp. at baseline (T0) between HOMA-ir Responders (>10 % decrease, *n* = 8) and Non-Responders (>10 % increase) (C–E) Differences in changes in subjects with low (*n* = 14) or high (*n* = 14) baseline *Blautia* spp. relative abundance in the treatment group in (C) CV% as a measure of glycaemic control (low *n* = 13, high *n* = 14; repeated-measures ANOVA with main effect of period *p* < 0.001, post-hoc tests with FDR-adjustment), (D) fasting glucose levels and (E) HOMA-ir as glucose homeostasis markers (assessed using non-parametric testing). ^‡^*p* < 0.05, ^‡‡^*p* < 0.01.

In recent years continuous glucose monitoring (CGM) has been developed to provide clinically relevant and dynamic insights into glucose profiles next to static biomarkers (Danne et al., 2017). Hence, we also monitored continuous glucose levels at baseline, during run-in and during the study period on three consecutive days to understand the development of glycaemic control over the intervention, as assessed by the coefficient of variation (CV) of glucose levels (Danne et al., 2017; Peyser et al., 2018). We observed that the CV decreased from 21.3 ± 0.94 % at baseline to 18.1 ± 0.97 % during run-in (*p =* 0.001) and 18.3 ± 0.84 % in the study period (*p =* 0.004) in the treatment group. The CV also decreased to a lower extent in the placebo group from 20.6 ± 1.07 % to 18.7 ± 1.00 % (run-in, *p =* 0.026) and 18.9 ± 0.86 % (study, *p =* 0.065; Supplemental Figure S5), yet following a sensitivity analysis only the decreases in the treatment group was sustained (run-in *p =* 0.018 and study *p =* 0.024; Figure 3A and Supplemental Methods). These observations indicated that dried chicory root intake had the capacity to improve the dynamics of glucose levels over time.

### Effect of baseline *Blautia* spp. relative abundance on T2D biomarkers

Besides the general effect of dried chicory root on glucose homeostasis in subjects at risk of T2D, we wanted to understand how the gut microbiota potentially impacts changes in the biomarkers of glycaemic control. Hence, we further investigated the glucose homeostasis response by dividing the treatment group into HOMA-ir responders (>10 % decrease HOMA-ir, *n* = 8) and non-responders (>10 % increase HOMA-ir, *n* = 12). The most discriminative genus included acetogenic *Blautia* spp. and at baseline HOMA-ir responders had significantly lower relative *Blautia* spp. amounts (1.4-fold lower; *p* = 0.01, Figure 3B). Consequently, we segmented the intervention group based on median baseline abundance of *Blautia* spp. into low (*n* = 14) and high abundance (*n* = 14). Remarkably, we observed a decrease in fasting glucose levels of −5.1 % (−0.31 ± 0.11 mmol/L, *p* = 0.013) in the low *Blautia* group, while the high *Blautia* group remained unchanged (0.06 ± 0.10 mmol/L, *p* = 0.546) leading to a difference between groups of 0.38 ± 0.15 mmol/L (*p* = 0.019; Figure 3D). Following up on this observation, we also observed HOMA-ir decreased more pronouncedly with −10.8 % (−0.14 ± 0.10 HOMA-ir, *p* = 0.289) compared to the high *Blautia* group (+0.07 ± 0.12 HOMA-ir, *p* = 0.055), resulting in a difference of 0.21 ± 0.16 between groups (*p* = 0.045; Figure 3E). Similarly, fasting insulin levels decreased with −9.9 % (−0.82 ± 0.65 low *Blautia* vs. 0.45 ± 0.75 μU/mL high *Blautia*; *p* = 0.210; Supplemental Table S9).

Of note, segmenting subjects into those with low *Blautia* spp. baseline levels also led to even more pronounced effects on glucose variability and fasting circulating SCFA. Glucose variability decreased in the low *Blautia* group from 21.3 ± 1.3 to 17.1 ± 1.0 % in run-in (*p* = 0.002) and 17.7 ± 0.8 % during the study (*p* = 0.018), while in the high *Blautia* group this decrease was more gradual from 21.4 ± 1.4 to 19.0 ± 1.6 % in run-in (*p* = 0.127) and 18.3 ± 1.4 % during the study period (*p* = 0.061; Figure 3C). Total fasting circulating SCFA increased by +22.7 % nearly three-times more in the low *Blautia* group (+11.66 ± 5.64 μM; *p* = 0.059) compared to +8.0 % in the high *Blautia* group (+3.75 ± 5.32 μM; *p =* 0.493), which was associated with a higher increase in fasting acetate levels in the low *Blautia* (+10.26 ± 5.00 μM, *p =* 0.061) vs. the high *Blautia* group (+4.13 ± 5.30 μM, *p =* 0.450; Supplemental Figure S6A,B). Interestingly, fasting propionate levels increased only in the low *Blautia* group by +27.0 % (+1.39 ± 1.16 μM, *p =* 0.252) and fasting butyrate levels remained unchanged (−0.01 ± 0.9 μM, *p =* 0.688) while in the high *Blautia* group fasting propionate (−0.29 ± 0.73 μM, *p =* 0.702) and butyrate levels slightly decreased (−0.08 ± 0.19 μM, *p =* 0.813; Supplemental Figure S6C,D). Hence, modulation of metabolic markers by the dried chicory root intake was observed to relate to differences in the baseline abundance of gut bacteria.

## Discussion

We examined the effect of an intrinsic, high-fibre product based on dried chicory roots, consisting of native inulin, pectin, hemicellulose and cellulose embedded in plant cells, in adults at risk of T2D. We observed a significant and strong dose-dependent and reversible effect of the treatment on improved bowel function, microbiota composition and activity as well as noted improvement of dynamic glucose levels measured by CGM.

Purified native inulin is thought to stimulate bacterial growth in the colon, which leads to increased bacterial mass and faecal bulk, and is hence well-known for its positive effect on bowel function maintenance (EFSA, 2015). Here we observed that both stool softness and stool frequency increased after 15 g/day and further after 30 g/day dried chicory root treatment and returned to baseline levels after the wash-out, indicating a time- and dose-dependent effect of the treatment on the colonic environment by the intrinsic fibres, that by definition include native inulin within the plant cell walls.

Modulation of the gut microbiota towards the stimulation of taxa that are associated with health benefits forms the basis for prebiotic interventions (Gibson et al., 2017). Two well-known genera in this context are *Bifidobacterium* and *Anaerostipes* spp., which have been reported to be slightly increased (approximately 1.2–1.4-fold) following intake of isolated, purified ITF (Vandeputte et al., 2017). However, the strong overall modulation of the gut microbiota (Figure 2A–D) and the more than three-fold increase in both *Bifidobacterium* and *Anaerostipes* spp. we observed here (Figure 2E), is remarkable and much higher compared to earlier ITF studies. Especially in subjects with low baseline abundance of these genera, we observed an increase by up to 11.55-fold for *Bifidobacterium* and 6.12-fold for *Anaerostipes* spp. after 30 g/day treatment (Supplemental Table S5). One study in healthy subjects consuming inulin-rich foods instead of purified ITF for 2 weeks also observed a four-fold increase in *Bifidobacterium* spp. but no changes in *Anaerostipes* spp. (Hiel et al., 2019). The high co-occurring fold-changes observed with the dried chicory roots point towards an improvement of the trophic chain involving bacteria of these two genera. Several *Bifidobacterium* spp., including the here identified *B. longum*, are known to use inulin and ITF, generating acetate and lactate (Falony et al., 2009; Flint et al., 2012). These metabolites are proven substrates for butyrate production by all known *Anaerostipes* species such as the here detected *A. hadrus* and *A. butyraticus* (Allen-Vercoe et al., 2012; Bui et al., 2019, 2021). Butyrate production is a desired feature of microbial fibre degradation since this SCFA is known to maintain gut homeostasis, for example by serving as energy source for colonocytes (Canfora et al., 2015). However, the plant cell walls might shield inulin from utilisation by these two genera. To assess the possibility of a trophic chain from the dried chicory root we performed a proof-of-concept experiment using a minimal set of strains with the canonical functionality of the genera of interest. We needed to include a pectin-utilizer like *Bacteroides xylanisolvens* HMP (Despres et al., 2016) to fully unlock the potential of the dried chicory roots *in vitro. Bacteroides* spp. as well as *Prevotella* spp. are known degraders of plant cell wall fibres (Martens et al., 2011) that may partially degrade and thereby open up the plant cells, and both genera were are found to be abundantly present in faecal samples (Figure 2E and Supplemental Figure S3). Using the synthetic tri-culture consisting of selected strains of *Bacteroides, Bifidobacterium* and *Anaerostipe*s spp., we indeed observed a substantial increase in butyrate (Figure 2F), for which the presence of *A. rhamnosivorans* was essential as it consumed lactate and acetate generated by *Bacteroides* spp. and *Bifidobacterium* spp. and in small amounts present in the YCFA medium (Duncan et al., 2009). It is possible that *Anaerostipes* spp. may even form propionate from a small amount of inositol in chicory (Hernández-Hernández et al., 2011) that is converted via the recently discovered metabolic pathway in *A. rhamnosivorans* and some other *Anaerostipes* spp. (Bui et al., 2021). Based on these proof-of-concept *in vitro* simulations, we propose a multi-species microbial network, operating in the colon of the subjects consuming dried chicory roots, that generates butyrate and propionate (Figure 2G).

Fibre-derived colonic SCFA that subsequently enter the systemic circulation are believed to contribute to the preventive effect of fermentable fibres on metabolic diseases (Canfora et al., 2015). Here, we observed that intake of dried chicory root can increase all three SCFA, that is acetate, propionate and butyrate, by more than a quarter of their baseline levels (Table 2), which is much higher than previously reported in studies with purified ITF. Most ITF studies detected small or no differences (Baxter et al., 2019; Kiewiet et al., 2021; Vandeputte et al., 2017) or even a decrease in faecal SCFA (Salazar et al., 2015). One trial reported an increase in all three SCFA after 6 weeks of 16 g/day ITF supplementation by a total of about one sixth over baseline levels (Birkeland et al., 2020). However, the reported mean increase in butyrate was 15 %, which is lower than the here found increase by more than 19 % (Table 2) and the higher proportion of subjects with faecal butyrate increase (*p* = 0.015). Interestingly, the subjects in the trial already had a high baseline fibre intake of 32.2 g/day in contrast to that in our study (22.8 ± 7.9 g fibre/day, Table 1). Another trial using mainly whole foods instead of purified fibres to increase fibre intake did not detect changes in faecal SCFA levels after 2 weeks of 40–50 g/day fibre intake (Oliver et al., 2021). In the placebo group faecal SCFA levels slightly decreased (Table 2), an observation reported also in other fibre studies (Birkeland et al., 2020; Chambers et al., 2019; Deroover et al., 2021). Faecal SCFA levels are a reflection of the balance between SCFA production, uptake and potential use by colonocytes (Canfora et al., 2015). Isolated, purified inulin is a chemically and physically simple polymer, readily fermentable in the proximal colon (Flint et al., 2012). In contrast, in dried chicory roots the microbial degradation of native inulin and other fibres into SCFA is potentially slowed down due to the enclosure within plant cells, resulting in a gradual release (Puhlmann and de Vos, 2020). Therefore, we hypothesise that dried chicory root fermentation was shifted towards a more distal location leading to a higher recovery of faecal SCFA as compared to purified ITF (Dagbasi et al., 2020; Hansen and Sams, 2018). The physical location of SCFA uptake in the colon appears to be critical for metabolic health markers favouring a distal above a proximal SCFA uptake (van der Beek et al., 2016). Hence, such a shift in location of SCFA production could impact circulating SCFA levels and might be a distinguishing and desired therapeutic feature of intrinsic fibre products (Canfora et al., 2015; Müller et al., 2019; So et al., 2021; van der Beek et al., 2016).

The effect of SCFA reaching the systemic circulation is not often reported in fibre studies. Systemically available SCFA are ligands to GPR41 and GPR43 expressed on various organs involved in T2D aetiology (Canfora et al., 2015). Intriguingly, circulating rather than faecal SCFA have been related to markers of insulin sensitivity (Müller et al., 2019) pointing towards a potential benefit of increasing their levels. We observed that besides faecal SCFA, the intake of dried chicory root also increased fasting circulating SCFA by more than 14 % compared to placebo (Table 3). In contrast, a 7-week cross-over trial in overweight subjects with inulin and inulin-propionate reported lower total fasting SCFA (−9.0 %), acetate (−9.9 %) and butyrate (−9.1 %) levels than the cellulose-control, while propionate (+6.7 %) levels were higher (Chambers et al., 2019). Interestingly, a recent 4-week parallel trial with reduced sized wheat bran particles instead of purified fibre in subjects with obesity only showed a normalisation of circulating SCFA to levels of normal weight subjects but no effects on faecal microbiota or health parameters (Deroover et al., 2021). This contrasts strongly with our observations and although the nature of the subjects, fibre type and structure differ between the trials, it suggests that gut microbiota changes are prerequisite for SCFA-mediated health-promoting effects of dietary fibre modulations.

In view of the pronounced changes in faecal microbiota and metabolites, we observed only subtle changes in static glucose homeostasis markers (Supplemental Table S8). In another parallel, but longer, 24-week trial in overweight subjects, HOMA-ir decreased over baseline by −14.8 % (from 2.7 to 2.3), fasting insulin by −7.8 %, and fasting glucose by −2.0 % after ITF consumption (Chambers et al., 2015). However, these levels did also not differ from baseline, and no non-fermentable placebo was included. A 7-week cross-over design in overweight and obese patients found a post-intervention difference between ITF and cellulose-control of 1.17 versus 1.59 in HOMA-ir and 9.0 μU/mL versus 12.3 μU/mL in fasting insulin without differences in fasting glucose levels (Chambers et al., 2019). In comparison to the static plasma glucose and insulin concentrations that represent rather a snapshot than a dynamic response, we observed that the CV as a measure of glucose variability decreased during run-in and the study period below 20 % (Figure 3A). Glucose variability assessed by CGM has developed into an important clinical variable besides traditional glycaemic markers (Danne et al., 2017) and CV’s below 20 % are reported for non-diabetic adults (Peyser et al., 2018) and considered to reflect stable glucose control in diabetes treatment (Monnier et al., 2017). In contrast, a similar trial in length and design using 30 g/day purified ITF did not observe changes in glucose variability assessed by CGM, even though subjects were more insulin resistant than in our study (Guess et al., 2016). A decrease in dynamic glucose variability below 20 % after dried chicory root intake might point towards an improvement in glucose control, potentially mediated by circulating SCFA and not yet manifested in static glycaemic markers.

It has been reported in early studies that the baseline gut microbiota composition may affect the response to fibre interventions, allowing the stratification in responders and non-responders (Korpela et al., 2014; Salonen et al., 2014). This was also found in a recent ITF intervention on weight loss in an obesity cohort (Rodriguez et al., 2020). Similarly, a recent trial with wheat-bran arabinoxylan oligosaccharides indicated that baseline levels of *Prevotella* spp. affected the faecal microbiota response to the fibre intervention (Chung et al., 2020). However, we could not confirm a similar impact in the present intervention with the dried chicory root. In contrast, we observed here *Blautia* spp. to discriminate between subjects that responded to the treatment with a decrease in HOMA-ir versus those who did not (Figure 3B). This is noteworthy, since studies have found that *Blautia* spp. levels are increased in T2D and also T1D patients compared to healthy controls (Gurung et al., 2020; Qi et al., 2016). *Blautia* spp. has been implied as heritable risk factor for visceral fat mass predisposing to metabolic disease (Le Roy et al., 2017) and associated with long-term consumption of processed foods (Bolte et al., 2021). Some studies attributed beneficial properties to *Blautia* spp. (Benítez-Páez et al., 2020), which may be caused by the incorrect assumption that these species produce butyrate – this is not the case (Louis and Flint, 2017) and this group of intestinal acetogens might be undesired in the context of insulin sensitivity. Consequently, we segmented the treatment based on low (*n* = 14) and high (*n* = 14) relative abundance of *Blautia* spp. and remarkedly observed that static glycaemic markers pronouncedly decreased in the low *Blautia* group, but not in the high *Blautia* group (Figure 3D,E). Moreover, a low *Blautia* spp. baseline relative abundance also appeared to impact more pronouncedly the other metabolic markers as CV of glucose levels decreased faster (Figure 3C) and circulating SCFA were higher in the low *Blautia* group compared to the high *Blautia* group (Supplemental Figure S6). We identified as major human *Blautia* species in this study *B. hominis*, *B. luti* and *B. obeum.* Several of these species are abundant members in the human gut and utilise different sugars and starch to produce acetate (Liu et al., 2008; Shin et al., 2018; Touyama et al., 2015). It has been reported that subjects consuming processed foods have increased levels of *Blautia* spp. (Bolte et al., 2021; Koponen et al., 2021). Hence, it is possible that subjects with these dietary habits are initially less responsive to the intrinsic fibre intake. We observed a decrease in *Blautia* spp. levels after 30 g/day dried chicory root treatment (Figure 2E) and also an ITF-induced decrease in this genus has been reported earlier (Chambers et al., 2019; Hiel et al., 2020). Extrapolation of the intervention-induced decrease in relative amounts of *Blautia* spp. suggested the high *Blautia* group might reach levels of the low *Blautia* group after an additional 6–8 weeks. This is relevant since a recent meta-analysis concluded that ITF interventions of 6 weeks or longer are needed to sufficiently decrease T2D markers in diabetic subjects (Wang et al., 2019). This also addresses the most important limitations of this study, which includes the short intervention duration that precludes meaningful measuring of Hb1Ac levels and the rather low level of insulin resistance.

In conclusion, this study shows the rapid effect of intrinsic fibre intake on bowel function, gut microbiota composition, faecal and fasting circulating SCFA and glucose variability in subjects at risk of T2D. The evidence for a trophic chain including *Bifidobacterium* and *Anaerostipes* spp. was recapitulated by *in vitro* incubations that resulted in high levels of butyrate and propionate production from the treatment product. Moreover, we observed a simultaneous increase in faecal and circulating SCFA levels and a marked improvement in dynamic markers of glucose control (CGM). In subjects with a low relative abundance of *Blautia* spp. – a genus that previously has been associated with T2D – also static glycaemic markers decreased pronouncedly. Since the chicory root treatment decreased levels of *Blautia* spp. (Figure 2E), increasing the intervention time is expected to provide glucose homeostasis improvement for all subjects at risk for T2D. Our results demonstrate a strong modulatory potential on gut health and microbial metabolism by native inulin and cell wall fibres pectin, cellulose and hemicellulose in the intrinsic form of dried chicory roots. Incorporating these minimally-processed, intrinsic fibres into long-term dietary therapeutic interventions could greatly impact the management of metabolic health via the increased levels of faecal and circulating SCFA.

## Abbreviations

BSS: Bristol Stool Scale
CGM: continuous glucose monitoring
CV: coefficient of variation (%) assessed by CGM
ENA: European Nucleotide Archive
GPR: G-protein-coupled receptors
ITF: inulin-type fructans
PCoA: principal coordinate analysis
PERMANOVA: permutational multivariate analysis of variance
SCFA: short-chain fatty acids
T2D: type 2 diabetes
YCFA: medium yeast extract, casitone and fatty acid medium

## Supplementary material

For supplementary material accompanying this paper visit https://doi.org/10.1017/gmb.2022.4. click here to view supplementary material

## References

[r1] Allen-Vercoe E, Daigneault M, White A, Panaccione R, Duncan SH, Flint HJ, O’Neal L and Lawson PA (2012) *Anaerostipes hadrus* comb. nov., a dominant species within the human colonic microbiota; reclassification of *Eubacterium hadrum* Moore *et al*. 1976. Anaerobe 18(5), 523–529. 10.1016/j.anaerobe.2012.09.002 22982042

[r2] Alssema M, Feskens EJM, Bakker SJL, Gansevoort RT, Boer JMA, Heine RJ, Nijpels G, Stehouwer CDA, Van Der Kraan M and Dekker JM (2008) Finse vragenlijst redelijk goede voorspeller van het optreden van diabetes in Nederland. Nederlands Tijdschrift voor Geneeskunde 152(44), 2418–2424. 19055143

[r3] American Diabetes Association (2016). 2. Classification and diagnosis of diabetes. Diabetes Care 39(Suppl. 1), S13–S22. 10.2337/dc16-S005 26696675

[r4] Augustin LSA, Aas A-M, Astrup A, Atkinson FS, Baer-Sinnott S, Barclay AW, Brand-Miller JC, Brighenti F, Bullo M, Buyken AE, Ceriello A, Ellis PR, Ha M-A, Henry JC, Kendall CWC, La Vecchia C, Liu S, Livesey G, Poli A, Salas-Salvadó J, Riccardi G, Riserus U, Rizkalla SW, Sievenpiper JL, Trichopoulou A, Usic K, Wolever TMS, Willett WC, Jenkins DJA (2020) Dietary fibre consensus from the international carbohydrate quality consortium (ICQC). Nutrients 12(9), 2553. 10.3390/nu12092553 32846882 PMC7551906

[r5] Bates D, Mächler M, Bolker B and Walker S (2015) Fitting linear mixed-effects models using lme4. Journal of Statistical Software 67(1), 1–48. 10.18637/JSS.V067.I01

[r6] Baxter NT, Schmidt AW, Venkataraman A, Kim KS, Waldron C and Schmidt TM (2019) Dynamics of human gut microbiota and short-chain fatty acids in response to dietary interventions with three fermentable fibers. MBio 10(1), e02566–e02518. 10.1128/mBio.02566-18 30696735 PMC6355990

[r7] Benítez-Páez A, Gómez del Pugar EM, López-Almela I, Moya-Pérez Á, Codoñer-Franch P and Sanz Y (2020) Depletion of Blautia species in the microbiota of obese children relates to intestinal inflammation and metabolic phenotype worsening. MSystems 5(2), e00857–e00819. 10.1128/mSystems.00857-19 32209719 PMC7093825

[r8] Birkeland E, Gharagozlian S, Birkeland KI, Valeur J, Måge I, Rud I and Aas A-M (2020) Prebiotic effect of inulin-type fructans on faecal microbiota and short-chain fatty acids in type 2 diabetes: A randomised controlled trial. European Journal of Nutrition 59(7), 3325–3338. 10.1007/s00394-020-02282-5 32440730 PMC7501097

[r9] Bolte LA, Vich Vila A, Imhann F, Collij V, Gacesa R, Peters V, Wijmenga C, Kurilshikov A, E Campmans-Kuijpers MJ, Fu J, Dijkstra G, Zhernakova A and Weersma RK (2021) Gut microbiota long-term dietary patterns are associated with pro-inflammatory and anti-inflammatory features of the gut microbiome. Gut 70(7), 1287–1298. 10.1136/gutjnl-2020-322670 33811041 PMC8223641

[r10] Bottacini F, Dal Bello F, Turroni F, Milani C, Duranti S, Foroni E, Viappiani A, Strati F, Mora D, van Sinderen D and Ventura M (2011) Complete genome sequence of Bifidobacterium animalis subsp. lactis BLC1. Journal of Bacteriology 193(22), 6387–6388. 10.1128/JB.06079-11 22038957 PMC3209193

[r11] Bui TPN, de Vos WM and Plugge CM (2014) *Anaerostipes rhamnosivorans* sp. nov., a human intestinal, butyrate-forming bacterium. International Journal of Systematic and Evolutionary Microbiology 64(Pt 3), 787–793. 10.1099/ijs.0.055061-0 24215821

[r12] Bui TPN, Mannerås-Holm L, Puschmann R, Wu H, Troise AD, Nijsse B, Boeren S, Bäckhed F, Fiedler D and DeVos WM (2021) Conversion of dietary inositol into propionate and acetate by commensal *Anaerostipes* associates with host health. Nature Communications, 12(1), 1–16. 10.1038/s41467-021-25081-w PMC8355322 34376656

[r13] Bui TPN, Schols HA, Jonathan M , Stams AJM, de Vos WM and Plugge CM (2019) Mutual metabolic interactions in co-cultures of the intestinal *Anaerostipes rhamnosivorans* with an acetogen, methanogen, or pectin-degrader affecting butyrate production. Frontiers in Microbiology 10, 2449. 10.3389/fmicb.2019.02449 31736896 PMC6839446

[r14] Canfora EE, Jocken JWE and Blaak EE (2015) Short-chain fatty acids in control of body weight and insulin sensitivity. Nature Reviews Endocrinology 11(10), 577–591. 10.1038/nrendo.2015.128 26260141

[r15] Canfora EE, van der Beek CM, Jocken JWE, Goossens GH, Holst JJ, Olde Damink SWM, Lenaerts K, Dejong CHC and Blaak EE (2017) Colonic infusions of short-chain fatty acid mixtures promote energy metabolism in overweight/obese men: A randomized crossover trial. Scientific Reports 7(1), 2360. 10.1038/s41598-017-02546-x 28539646 PMC5443817

[r16] Chambers ES, Byrne CS, Morrison DJ, Murphy KG, Preston T, Tedford C , Garcia-Perez I, Fountana S, Serrano-Contreras JI, Holmes E, Reynolds CJ, Roberts JF, Boyton RJ, Altmann DM, Mcdonald JAK, Marchesi JR, Akbar AN, Riddell NE, Wallis GA and Frost GS (2019) Dietary supplementation with inulin-propionate ester or inulin improves insulin sensitivity in adults with overweight and obesity with distinct effects on the gut microbiota, plasma metabolome and systemic inflammatory responses: A randomised cross-over trial. Gut 68(8), 1430–1438. 10.1136/gutjnl-2019-318424 30971437 PMC6691855

[r17] Chambers ES, Viardot A, Psichas A, Morrison DJ, Murphy KG, Zac-Varghese SEK, MacDougall K, Preston T, Tedford C, Finlayson GS, Blundell JE, Bell JD, Thomas EL, Mt-Isa S, Ashby D, Gibson GR, Kolida S, Dhillo WS, Bloom SR, Morley W, Clegg S, Frost G (2015) Effects of targeted delivery of propionate to the human colon on appetite regulation, body weight maintenance and adiposity in overweight adults. Gut 64(11), 1744–1754. 10.1136/gutjnl-2014-307913 25500202 PMC4680171

[r18] Chung WSF, Walker AW, Bosscher D, Garcia-Campayo V, Wagner J, Parkhill J, Duncan SH and Flint HJ (2020) Relative abundance of the *Prevotella* genus within the human gut microbiota of elderly volunteers determines the inter-individual responses to dietary supplementation with wheat bran arabinoxylan-oligosaccharides. BMC Microbiology 20(1), 1–14. 10.1186/s12866-020-01968-4 32928123 PMC7490872

[r19] Dagbasi A, Lett AM, Murphy K and Frost G (2020) Understanding the interplay between food structure, intestinal bacterial fermentation and appetite control. Proceedings of the Nutrition Society 79(4), 1–17. 10.1017/S0029665120006941 32383415

[r20] Danne T, Nimri R, Battelino T, Bergenstal RM, Close KL, DeVries JH, Garg S, Heinemann L, Hirsch I, Amiel SA, Beck R, Bosi E, Buckingham B, Cobelli C, Dassau E, Doyle FJ, Heller S, Hovorka R, Jia W, Jones T, Kordonouri O, Kovatchev B, Kowalski A, Laffel L, Maahs D, Murphy HR, Nørgaard K, Parkin CG, Renard E, Saboo B, Scharf M, Tamborlane WV, Weinzimer SA, Phillip M (2017) International consensus on use of continuous glucose monitoring. Diabetes Care 40(12), 1631–1640. 10.2337/DC17-1600 29162583 PMC6467165

[r21] De Vos WM and Nieuwdorp M (2013) Genomics: A gut prediction. Nature 498(7452), 48–49. 10.1038/nature12251 23719383

[r22] Deroover L, Vázquez-Castellanos JF, Vandermeulen G, Luypaerts A, Raes J, Courtin CM and Verbeke K (2021) Wheat bran with reduced particle size increases serum SCFAs in obese subjects without improving health parameters compared with a maltodextrin placebo. The American Journal of Clinical Nutrition 114, 1328–1341. 10.1093/ajcn/nqab196 34224554

[r23] Despres J, Forano E, Lepercq P, Comtet-Marre S, Jubelin G, Yeoman CJ, Miller MEB, Fields CJ, Terrapon N, Bourvellec C, Renard CMGC, Henrissat B, White BA and Mosoni P (2016) Unraveling the pectinolytic function of *Bacteroides xylanisolvens* using a RNA-seq approach and mutagenesis. BMC Genomics 17(1), 147. 10.1186/s12864-016-2472-1 26920945 PMC4769552

[r24] Dewulf EM, Cani PD, Claus SP, Fuentes S, Puylaert PG, Neyrinck AM, Bindels LB, de Vos WM, Gibson GR, Thissen JP and Delzenne NM (2013) Insight into the prebiotic concept: Lessons from an exploratory, double blind intervention study with inulin-type fructans in obese women. Gut, 62(8), 1112–1121. 10.1136/gutjnl-2012-303304 23135760 PMC3711491

[r25] Duncan SH, Louis P, Thomson JM and Flint HJ (2009) The role of pH in determining the species composition of the human colonic microbiota. Environmental Microbiology 11(8), 2112–2122. 10.1111/j.1462-2920.2009.01931.x 19397676

[r26] EFSA (2015) Scientific opinion on the substantiation of a health claim related to “native chicory inulin” and maintenance of normal defecation by increasing stool frequency pursuant to article 13.5 of regulation (EC) no 1924/2006. EFSA Journal 13(1), 3951. 10.2903/j.efsa.2015.3951

[r27] Falony G, Lazidou K, Verschaeren A, Weckx S, Maes D and De Vuyst L (2009) In vitro kinetic analysis of fermentation of prebiotic inulin-type fructans by *Bifidobacterium* species reveals four different phenotypes. Applied and Environmental Microbiology 75(2), 454–461. 10.1128/AEM.01488-08 19011052 PMC2620708

[r28] Fan Y and Pedersen O (2020) Gut microbiota in human metabolic health and disease. Nature Reviews Microbiology 19(1), 55–71. 10.1038/s41579-020-0433-9 32887946

[r29] Flint HJ, Scott KP, Duncan SH, Louis P and Forano E (2012) Microbial degradation of complex carbohydrates in the gut. Gut Microbes 3(4), 289–306. 10.4161/gmic.19897 22572875 PMC3463488

[r30] Gibson GR, Hutkins R, Sanders ME, Prescott SL, Reimer RA, Salminen SJ, Scott K, Stanton C, Swanson K. S, Cani PD, Verbeke K and Reid G (2017) Expert consensus document: The International Scientific Association for Probiotics and Prebiotics (ISAPP) consensus statement on the definition and scope of prebiotics. Nature Reviews Gastroenterology & Hepatology 14(8), 491–502. 10.1038/nrgastro.2017.75 28611480

[r31] Grundy MML, Edwards CH, Mackie AR, Gidley MJ, Butterworth PJ and Ellis PR (2016) Re-evaluation of the mechanisms of dietary fibre and implications for macronutrient bioaccessibility, digestion and postprandial metabolism. British Journal of Nutrition 116(5), 816–833. 10.1017/S0007114516002610 27385119 PMC4983777

[r32] Guess ND, Dornhorst A, Oliver N and Frost GS (2016) A randomised crossover trial: The effect of inulin on glucose homeostasis in subtypes of prediabetes. Annals of Nutrition and Metabolism 68(1), 26–34. 10.1159/000441626 26571012

[r33] Gurung M, Li Z, You H, Rodrigues R, Jump DB, Morgun A and Shulzhenko N (2020) Role of gut microbiota in type 2 diabetes pathophysiology. EBioMedicine 51, 102590. 10.1016/j.ebiom.2019.11.051 31901868 PMC6948163

[r34] Hansen NW and Sams A (2018) The microbiotic highway to health-new perspective on food structure, gut microbiota, and host inflammation. Nutrients 10(11), 1590. 10.3390/nu10111590 30380701 PMC6267475

[r35] Hernández-Hernández O, Ruiz-Aceituno L, Sanz ML and Martínez-Castro I (2011) Determination of free Inositols and other low molecular weight carbohydrates in vegetables. Journal of Agricultural and Food Chemistry 59(6), 2451–2455. 10.1021/JF1045552 21366313

[r36] Hiel S, Bindels LB, Pachikian BD, Kalala G, Broers V, Zamariola G, Chang BPI, Kambashi B, Rodriguez J, Cani PD, Neyrinck AM, Thissen J-P, Luminet O, Bindelle J and Delzenne NM (2019) Effects of a diet based on inulin-rich vegetables on gut health and nutritional behavior in healthy humans. The American Journal of Clinical Nutrition 109(6), 1683–1695. 10.1093/ajcn/nqz001 31108510 PMC6537941

[r37] Hiel S, Gianfrancesco MA, Rodriguez J, Portheault D, Leyrolle Q, Bindels LB, Gomes da Silveira Cauduro C, Mulders MDGH, Zamariola G, Azzi AS, Kalala G, Pachikian BD, Amadieu C, Neyrinck AM, Loumaye A, Cani PD, Lanthier N, Trefois P, Klein O, Luminet O, Bindelle J, Paquot N, Cnop M, Thissen JP, Delzenne NM (2020) Link between gut microbiota and health outcomes in inulin -treated obese patients: Lessons from the Food4Gut multicenter randomized placebo-controlled trial. Clinical Nutrition 39(12), 3618–3628. 10.1016/j.clnu.2020.04.005 32340903

[r38] Jones JM (2014) CODEX-aligned dietary fiber definitions help to bridge the ‘fiber gap.’ Nutrition Journal 13(1), 34. 10.1186/1475-2891-13-34 24725724 PMC4007020

[r39] Kassambara A (2021) *rstatix: Pipe-Friendly Framework for Basic Statistical Tests. R package version 0.7.0.* 30 August 2021 Available at https://cran.r-project.org/package=rstatix.

[r40] Kiewiet MBG, Elderman ME, El Aidy S, Burgerhof JGM, Visser H, Vaughan EE, Faas MM and de Vos P (2021) Flexibility of gut microbiota in ageing individuals during dietary fiber long-chain inulin intake. Molecular Nutrition and Food Research 65(4), 2000390. 10.1002/mnfr.202000390 33369019 PMC8138623

[r41] Koponen KK, Salosensaari A, Ruuskanen MO, Havulinna AS, Männistö S, Jousilahti P, Palmu J, Salido R, Sanders K, Brennan C, Humphrey GC, Sanders JG, Meric G, Cheng S, Inouye M, Jain M, Niiranen TJ, Valsta LM, Knight R and Salomaa VV (2021) Associations of healthy food choices with gut microbiota profiles. The American Journal of Clinical Nutrition 114(2), 605–616. 10.1093/ajcn/nqab077 34020448 PMC8326043

[r42] Korpela K (2016) *mare: Microbiota Analysis in R Easily. R package version 1.0.* 10.5281/zenodo.50310

[r43] Korpela K, Flint HJ, Johnstone AM, Lappi J, Poutanen K, Dewulf E, Delzenne N, de Vos WM and Salonen A (2014) Gut microbiota signatures predict host and microbiota responses to dietary interventions in obese individuals. PLoS One 9(3), e90702. 10.1371/journal.pone.0090702 24603757 PMC3946202

[r44] Le Bastard Q., Chapelet G, Javaudin F, Lepelletier D, Batard E and Montassier E (2019) The effects of inulin on gut microbial composition: A systematic review of evidence from human studies. European Journal of Clinical Microbiology and Infectious Diseases 39(3), 403–413. 10.1007/s10096-019-03721-w 31707507

[r45] Le Roy CI, Beaumont M, Jackson MA, Steves CJ, Spector TD and Bell JT (2017) Heritable components of the human fecal microbiome are associated with visceral fat. Gut Microbes 9(1), 1–7. 10.1080/19490976.2017.1356556 28767316 PMC5914912

[r46] Lewis SJ and Heaton KW (1997) Stool form scale as a useful guide to intestinal transit time. Scandinavian Journal of Gastroenterology 32(9), 920–924. 10.3109/00365529709011203 9299672

[r47] Lindström J and Tuomilehto J (2003) The diabetes risk score: A practical tool to predict type 2 diabetes risk. Diabetes Care 26(3), 725–731. 10.2337/diacare.26.3.725 12610029

[r48] Liu C, Finegold SM, Song Y and Lawson PA (2008) Reclassification of *Clostridium coccoides, Ruminococcus hansenii, Ruminococcus hydrogenotrophicus, Ruminococcus luti, Ruminococcus productus* and *Ruminococcus schinkii* as B*lautia coccoides* gen. nov., comb. nov., *Blautia hansenii* comb. nov., *Blautia hydrogenotrophica* comb. nov., *Blautia luti* comb. nov., *Blautia producta* comb. nov., *Blautia schinkii* comb. nov. and description of *Blautia wexlerae* sp. nov., isolated from human faeces. International Journal of Systematic and Evolutionary Microbiology 58(8), 1896–1902. 10.1099/IJS.0.65208-0 18676476

[r49] Louis P and Flint HJ (2017) Formation of propionate and butyrate by the human colonic microbiota. Environmental Microbiology 19(1), 29–41. 10.1111/1462-2920.13589 27928878

[r50] Martens EC, Lowe EC, Chiang H, Pudlo NA, Wu M, McNulty NP, Abbott DW, Henrissat B, Gilbert HJ, Bolam DN and Gordon JI (2011). Recognition and degradation of plant cell wall polysaccharides by two human gut symbionts. PLoS Biology 9(12), e100121. 10.1371/journal.pbio.1001221 PMC3243724 22205877

[r51] Monnier L, Colette C, Wojtusciszyn A, Dejager S, Renard E, Molinari N and Owens DR (2017) Toward defining the threshold between low and high glucose variability in diabetes. Diabetes Care 40(7), 832–838. 10.2337/dc16-1769 28039172

[r52] Müller M, Hernández MAG, Goossens GH, Reijnders D, Holst JJ, Jocken JWE, van Eijk H, Canfora EE and Blaak EE (2019) Circulating but not faecal short-chain fatty acids are related to insulin sensitivity, lipolysis and GLP-1 concentrations in humans. Scientific Reports 9, 12515. 10.1038/s41598-019-48775-0 31467327 PMC6715624

[r53] Oliver A, Chase AB, Weihe C, Orchanian SB, Riedel SF, Hendrickson CL, Lay M, Sewall JM, Martiny JBH and Whiteson K (2021) High-fiber, whole-food dietary intervention alters the human gut microbiome but not fecal short-chain fatty acids. MSystems 6(2), e00115–e00121. 10.1128/msystems.00115-21 33727392 PMC8546969

[r54] Peyser TA, Balo AK, Buckingham BA, Hirsch IB and Garcia A (2018) Glycemic variability percentage: A novel method for assessing glycemic variability from continuous glucose monitor data. Diabetes Technology & Therapeutics 20(1), 6–16. 10.1089/DIA.2017.0187 29227755 PMC5846572

[r55] Puhlmann M-L and de Vos WM (2020) Back to the roots: Revisiting the use of the fiber-rich *Cichorium intybus* L. Taproots. Advances in Nutrition 11(4), 878–889. 10.1093/advances/nmaa025 32199025 PMC7360457

[r56] Qi C-J, Zhang Q, Yu M, Xu J-P, Zheng J, Wang T and Xiao X-H (2016) Imbalance of fecal microbiota at newly diagnosed type 1 diabetes in Chinese children. Chinese Medical Journal 129(11), 1298–1304. 10.4103/0366-6999.182841 27231166 PMC4894039

[r57] Reynolds A, Akerman A and Mann J (2020) Dietary fibre and whole grains in diabetes management: Systematic review and meta-analyses. PLoS Medicine 17(3), e1003053. 10.1371/journal.pmed.1003053 32142510 PMC7059907

[r58] Reynolds A, Mann J, Cummings J, Winter N, Mete E and Te Morenga L (2019) Carbohydrate quality and human health: A series of systematic reviews and meta-analyses. The Lancet 393(10170), 434–445. 10.1016/S0140-6736(18)31809-9 30638909

[r59] Rodriguez J, Hiel S, Neyrinck AM, Roy TL, Pötgens SA, Leyrolle Q, Pachikian BD, Gianfrancesco MA, Cani PD, Paquot N, Cnop M, Lanthier N, Thissen J-P, Bindels LB and Delzenne NM (2020) Discovery of the gut microbial signature driving the efficacy of prebiotic intervention in obese patients. Gut 69(11), 1975–1987. 10.1136/gutjnl-2019-319726 32041744 PMC7569399

[r60] Salazar N, Dewulf EM, Neyrinck AM, Bindels LB, Cani PD, Mahillon J, de Vos WM, Thissen J-P, Gueimonde M, de los Reyes-Gavilán CG and Delzenne NM (2015) Inulin-type fructans modulate intestinal Bifidobacterium species populations and decrease fecal short-chain fatty acids in obese women. Clinical Nutrition 34(3), 501–507. 10.1016/j.clnu.2014.06.001 24969566

[r61] Salonen A, Lahti L, Salojärvi J, Holtrop G, Korpela K, Duncan SH, Date P, Farquharson F, Johnstone AM, Lobley GE, Louis P, Flint HJ and De Vos WM (2014) Impact of diet and individual variation on intestinal microbiota composition and fermentation products in obese men. ISME Journal 8(11), 2218–2230. 10.1038/ismej.2014.63 24763370 PMC4992075

[r62] Shin N-R, Kang W, Tak EJ, Hyun D-W, Kim PS, Kim HS, Lee J-Y, Sung H, Whon TW and Bae J-W (2018) *Blautia hominis* sp. nov., isolated from human faeces. International Journal of Systematic and Evolutionary Microbiology 68(4), 1059–1064. 10.1099/ijsem.0.002623 29458493

[r63] So D, Gibson PR, Muir JG and Yao CK (2021) Dietary fibres and IBS: Translating functional characteristics to clinical value in the era of personalised medicine. Gut 70(12), 2383–2394. 10.1136/gutjnl-2021-324891 34417199

[r64] So D, Whelan K, Rossi M, Morrison M, Holtmann G, Kelly JT, Shanahan ER, Staudacher HM and Campbell KL (2018) Dietary fiber intervention on gut microbiota composition in healthy adults: A systematic review and meta-analysis. American Journal of Clinical Nutrition 107(6), 965–983. 10.1093/ajcn/nqy041 29757343

[r65] Swanson KS, De Vos WM, Martens EC, Gilbert JA, Menon RS, Soto-Vaca A, Hautvast J, Meyer PD, Borewicz K, Vaughan EE and Slavin JL (2020) Effect of fructans, prebiotics and fibres on the human gut microbiome assessed by 16S rRNA-based approaches: A review. Beneficial Microbes 11(2), 101–129. 10.3920/BM2019.0082 32073295

[r66] Touyama M, Jin JS, Kibe R, Hayashi H and Benno Y (2015) Quantification of Blautia wexlerae and Blautia luti in human faeces by real-time PCR using specific primers. Beneficial Microbes 6(4), 583–590. 10.3920/BM2014.0133 25691104

[r67] van der Beek CM, Canfora EE, Lenaerts K, Troost FJ, Damink S, Holst JJ, Masclee AAM, Dejong CHC and Blaak EE (2016) Distal, not proximal, colonic acetate infusions promote fat oxidation and improve metabolic markers in overweight/obese men. Clinical Science 130(22), 2073–2082. 10.1042/cs20160263 27439969

[r68] Vandeputte D, Falony G, Vieira-Silva S, Wang J, Sailer M, Theis S, Verbeke K and Raes J (2017) Prebiotic inulin-type fructans induce specific changes in the human gut microbiota. Gut 66(11), 1968–1974. 10.1136/gutjnl-2016-313271 28213610 PMC5739857

[r69] Wang L, Yang H, Huang H, Zhang C, Zuo HX, Xu P, Niu YM and Wu SS (2019) Inulin-type fructans supplementation improves glycemic control for the prediabetes and type 2 diabetes populations: Results from a GRADE-assessed systematic review and dose-response meta-analysis of 33 randomized controlled trials. Journal of Translational Medicine 17(1), 410. 10.1186/s12967-019-02159-0 31805963 PMC6896694

[r70] Wu H, Tremaroli V, Schmidt C, Lundqvist A, Olsson LM, Krämer M, Gummesson A, Perkins R, Bergström G and Bäckhed F (2020) The gut microbiota in prediabetes and diabetes: A population-based cross-sectional study. Cell Metabolism 32(3), 379–390.e3. 10.1016/j.cmet.2020.06.011 32652044

[r71] Zhao L, Zhang F, Ding X, Wu G, Lam YY, Wang X, Fu H, Xue X, Lu C, Ma J, Yu L, Xu C, Ren Z, Xu Y, Xu S, Shen H, Zhu X, Shi Y, Shen Q, Dong W, Liu R, Ling Y, Zeng Y, Wang X, Zhang Q, Wang J, Wang L, Wu Y, Zeng B, Wei H, Zhang M and Peng Y, Zhang C (2018) Gut bacteria selectively promoted by dietary fibers alleviate type 2 diabetes. Science 359(6380), 1151–1156. 10.1126/science.aao5774 29590046

