# Supplementary material for: Dried chicory root improves bowel function, benefits intestinal microbial trophic chains and increases faecal and circulating short chain fatty acids in subjects at risk for type 2 diabetes
Source: Gut Microbiome (Camb). 2022 Apr 28;3:e4. doi: 10.1017/gmb.2022.4 (PMC11407914; doi:10.1017/gmb.2022.4)
Supplement: Supplementary file 1 [file S2632289722000044sup001.pdf]

SUPPLEMENTARY MATERIAL

1. SUPPLEMENTARY METHODS

1.1 Trial design

Participants arrived at the first study visit after an overnight fast (minimum eight hours fasted), where the pre-intervention (T0) blood sample was drawn, and anthropometric measures were taken. Then the first sensor for the Continuous Glucose Monitoring (CGM) was placed. The study procedure was explained along with handing out the diaries for the bowel function and (gastrointestinal) well-being and sachets for the run-in period. At home, participants filled in the study diary for the past week, collected the first faecal sample (stored directly in-home freezer) and measured their glucose levels using the CGM. Three days after the first study visit, participants started consuming the treatment or placebo. At the end of the run-in period (T1), prior to the second study visit, participants collected the second faecal sample. On the second study visit, participants returned the faecal samples in frozen form and the diaries of the baseline and run-in period along with the empty leftover sachets of the run-in period. Anthropometric measures were taken again, the CGM sensor was replaced and new diaries along with the sachets for the intervention period were handed out. At the end of the intervention period (T2) participants collected the third faecal sample. At the third study visit participants arrived again fasted and the post-intervention blood sample was taken as well as anthropometric measures. The second CGM was removed, and the diaries of the intervention period and the empty and leftover sachets were returned. Participants received the diaries of the wash-out period. At the end of the wash-out period (T3) participants collected the fourth faecal sample, after which the third

and fourth faecal sample were transported to the university together with the diaries of the wash-out period.

The products were provided as single, daily dosage packed in transparent sachets stored in nontransparent bags for each week of the study. Intervention products were provided by a third, independent researcher. Neither the researchers nor the participants were told which intervention product they received. As the placebo product could not fully match the treatment product in colour, taste and shape, implying the possibility of participants to being not fully blinded, the study was considered investigator-blinded. Participants were instructed to consume the content of one sachet per day, preferably in the morning with their breakfast or morning snack. Compliance was assessed by counting empty and leftover sachets. All participants were instructed to maintain their normal diet and level of physical activity throughout the whole study.

1.2 Short-chain fatty analysis in faeces

Faecal samples were collected at home by the volunteers using sterile tubes and directly frozen in the home freezer at -20 °C. The samples were transported in frozen form within one month to the study centre where they were further stored at -80 °C. Quantification of faecal SCFA was done by analysing faecal water using high-performance chromatography (HPLC) similar to previously described methods (Bui et al., 2019). In short, faecal samples were diluted in water by mixing 0.2 g faeces with 0.8 mL water and subsequently centrifuged for 5 min at 21130 RCF at 4 °C. Standards of acetic acid, butyric acid, propionic acid, isobutyric acid and lactate were prepared in concentrations ranging from 2.5-20 mM. Then, 160 µL of standard or sample was mixed with 40 µL of 10 mM DSMO in 0.1 N H_2SO_4 as internal standard for analysis. SCFA were quantified using high-performance liquid

chromatography on a Shimadzu LC_2030C equipped with a refractive index detector and a Shodex SH1011 column. Ten µL of the sample was injected at an oven temperature of 45 °C with a flow rate of 1.00 mL/min using 0.01 N H_2SO_4 as eluent.

1.3 Diaries for bowel function and well-being

Participants were asked to record their bowel function and (gastrointestinal) well-being using weekly questionnaires. Bowel function was assessed as average stool softness over the last week using the Bristol Stool Scale and stool frequency as average number of stools per day during the last week. Gastrointestinal symptoms and satiety feelings were recorded using a 0 - 100 visual analogue scale.

1.4 Gut microbiota analysis.

Bacterial DNA was extracted from frozen faecal samples that were thawed on ice, using the repeated bead-beating method as described before (Salonen et al., 2010) and purified using the Maxwell® 16 Tissue LEV DNA Purification Kit. The quantity of the extracted DNA was assessed using a Nanodrop spectrophotometer (Thermo Fisher Scientific). Microbiota composition was analysed by Illumina MiSeq sequencing of the 16S rRNA gene hypervariable V3-V4 region using primers 341F/785R as previously described (Korpela et al., 2018). Sequences were further processed using the mare package in R (Korpela, 2016) that relies on USEARCH (Edgar, 2010). In short, only forward reads were used, primers were cut from the 5'-end of each read, and reads were quality filtered with quality score 2 and truncated to a length of 150 bp. The rarest reads, likely sequencing errors, were removed based on a minimum read abundance of 0.005%. After quality filtering, we obtained on average 34952 reads per sample, ranging from 3164 to 78916. The reads were taxonomically annotated

using USEARCH (Edgar, 2010) to map to the SILVA 16S rRNA reference database version 115 (Quast et al., 2013). OTUs (operational taxonomic unit), with clustering at 97% identity, were used to calculate the diversity and richness measures. Further analyses were performed using the mare package. The data has been submitted to the European Nucleotide Archive (ENA) under the accession number PRJEB47230. To attain species level annotations, the same reads were annotated using the BLAST function (Camacho et al., 2009) in the mare package.

1.5 Trophic chain *in vitro* experiment - synthetic co- and tri-cultures

As a proof-of-concept a trophic chain experiment with dried chicory root (WholeFiber™) was performed using synthetic co- and tri-cultures with strains having the canonical functionality of the genera of interest. For this purpose *Bifidobacterium animalis subsp. lactis* BLC1 (obtained from Dr L Morelli Sacco SRL), *Anaerostipes rhamnosivorans* $1Y2^T$ (Laboratory of Microbiology, Wageningen University & Research) and *Bacteroides xylanisolvens* HMP (obtained from the Human Microbiome Program as HMP 2_1_22) were grown in co- or tricultures. Bacteria were routinely maintained in a modified yeast extract, casitone fatty acid (YCFA) medium (Duncan et al., 2009) supplemented with 20 mM xylose and 20 mM galactose for *Bacteroides xylanisolvens* HMP or 20 mM glucose for *Bifidobacterium animalis subsp. lactis* BLC1 and *Anaerostipes rhamnosivorans* $1Y2^T$. All growth experiments were performed in duplicate in the modified YCFA medium (Duncan et al., 2009) containing 5 g/L WholeFiber™. Equal amounts of the overnight preculture of the three individual bacteria (5%, v/v) were simultaneously added to the media to complete the tri-culture. In parallel, same amounts of *Bacteroides xylanisolvens* HMP and *Bifidobacterium animalis subsp. lactis* BLC1 were added in the modified YCFA medium with dried chicory roots. All cultures were

subsequently incubated at 37°C up to seven days. Gas and liquid samples were collected at different time intervals for H_2 and organic acid analysis, respectively, as previously described (Bui et al., 2019).

1.6 Circulating short-chain fatty analysis in plasma

Fasting blood samples were collected after antecubital venepuncture into BD® EDTA K2E vacutainers (4 mL). Samples were centrifuged for 10 min at 4 °C and 12,000 x g and subsequently stored at -80 °C until further analysis. Fasting circulating short-chain fatty acids in plasma were derivatized prior to measurement on a liquid-chromatography triple quadrupole mass-spectrometry (LC-MS/MS) as previously described (Han et al., 2015) and optimized (van Dongen et al., 2021). The protocol was further adapted as described in the following. All chemicals were prepared at room temperature (RT). Plasma samples were first centrifuged for 5 min at RT and 14,000 x g. The supernatant was spiked with a mix of each 1 mM deuterated acetate (acetate-d_4) and butyrate (butyrate-d_7) by adding 5 µL of the deuterated SCFA mix to 45 µL of sample to achieve a concentration of 0.1 mM in the sample. The sample was subsequently cleaned by adding 50 µL spiked sample to 150 µL 100% acetonitrile (ratio 1:3) at RT and mixing by inversion. Subsequently, samples were centrifuged for 20 min at 14,000 x g. A volume of 100 µL of the supernatant was transferred in a low-binding Eppendorf tube to which 100 µL 200 mM 3-nitrophenylhydrazine (3NPH) in 40% acetonitrile and 100 µL 120 mM 1-ethyl-3-(3-dimethylaminopropyl) carbodiimide (EDC) with 6% pyridine in 40% acetonitrile were added. All reagents were prepared freshly in glass tubes at RT. After mixing by inversion the samples were derivatized by heating at 40 °C for 30 min while shaking in a thermomixer. Subsequently, samples were cooled on ice for 1 min, 100 µL of ice-cold water was added and samples were mixed by inversion. Derivatized samples were centrifuged for 5 min at 14,000 x g. A volume of 90 µL of the sample was

transferred to a LC-MS/MS vial to which 10 µL of an internal standard was added, which consisted 0.15 mM acetate, 0.1 mM butyrate and 0.1 mM propionate derivatized using isotope labelled $^{13}C_6$-3NPH instead of 3NPH as described before (van Dongen et al., 2021). For quantification, a calibration curve was made by derivatizing known concentrations of acetate, butyrate and propionate in the range of 0-60 µM as described above, of which 90 µL was mixed with 10 µL of the internal standard as done for the samples. Samples were stored at 4 °C until measurement on the same or subsequent day using a Shimadzu Nexera XR LC-20AD XR UPLC system coupled to a Shimadzu LCMS-8050 triple quadruple mass spectrometer (Kyoto, Japan). 10 µL of sample were injected onto a Phenomenex C18 column (50 x 2.1 mM, 1.7 µm) at 40 °C. The mobile phase consisted of a gradient made from ultrapure water with 0.1% formic acid (v/v) (mobile phase A) and acetonitrile with 0.1% formic acid (v/v) (mobile phase B). The gradient started with 100% mobile phase A, reaching 70% mobile phase A at 6 min, and further decreasing to 30% mobile phase A at 8 min. At 9 min 0% mobile phase A was reached and was kept until 10 min, returning to 100% mobile phase A at 10.5 min and kept at these initial conditions up to 15 min. The LCMS-8050 was equipped with an electrospray ionization (ESI) source and used for multiple reaction monitoring (MRM) quantification in negative mode with the MRM transitions from precursor to product with corresponding retention times as given in **Table S1.** A nebulizing gas flow of 3 L/min was combined with a heating gas flow of 10 L/min, an interface temperature of 300 °C, a desolvation temperature of 526 °C, a desolvation line temperature of 250 °C and a heat block temperature of 400 °C. The drying gas flow was set at 10 L/min.

To quantify SCFA concentrations the peak areas of the calibration curve standards were divided by the peak area of their corresponding ^{13}C isotope-labelled SCFA. Concentrations of SCFA in the samples were then calculated in the same way by dividing each SCFA peak area

for its corresponding ^{13}C isotope-labelled internal standard peak area. The variation measured by the ratio of the spiked-in deuterated acetate and butyrate corrected for their corresponding ^{13}C isotope labelled SCFA was found to be 13% and 10%, respectively. As butyrate is partially consumed by enterocytes (Deleu et al., 2021), circulating butyrate levels were close to the quantification threshold and, in certain cases, negative (mostly in the placebo group after the study). Hence all negative values were set equal to zero before further analysis and non-parametric distributions were assumed for the statistical analysis of this SCFA.

Table S1 Retention times and reaction monitoring transitions for detection of the derivatized short chain fatty acids by liquid-chromatography triple quadrupole mass-spectrometry.

**Short-chain fatty acid**	**Retention time (min)**	**Precursor ion *(m/z)***	**Product ion *(m/z)***	**Collision energy (V)**
Acetate 3NPH	4.35	194.0	137.1	17.0
Acetate $^{13}C_6$-3NPH	4.35	200.1	143.1	17.0
Acetate-d_4 3NPH	4.32	197.1	137.1	17.0
Propionate 3NPH	5.41	208.0	137.1	19.0
Propionate $^{13}C_6$-3NPH	5.41	214.1	143.1	19.0
Butyrate 3NPH	6.54	222.1	137.1	19.0
Butyrate $^{13}C_6$-3NPH	6.54	228.1	143.1	19.0
Butyrate-d_7 3NPH	6.54	229.1	137.1	19.0

3NPH, 3-nitrophenylhydrazine

1.7 Glucose homeostasis markers

Fasting blood samples were collected after antecubital venepuncture into BD® sodium fluoride vacutainers (2 mL) for glucose measurement and into BD® EDTA K2E vacutainers (4 mL) for insulin. Samples were centrifuged for 10 min at 4 °C and 12,000 x g and

subsequently stored at -80 °C until further analysis. Fasting glucose concentrations were analysed in the automated systems of the local hospital "De Gelderse Vallei" (Ede, The Netherlands). Fasting insulin concentrations were measured with enzyme-linked immunosorbent assay (ELISA) (Mercodia Ultrasensitive Insulin ELISA, Uppsala, Sweden). HOMA-ir values were calculated from fasting glucose and fasting insulin values using the HOMA2 calculator (https://www.dtu.ox.ac.uk/homacalculator) (Levy et al., 1998; Matthews et al., 1985).

1.8 Continuous Glucose Monitoring

Abbott's FreeStyle Libre Flash was used for Continuous Glucose Monitoring (CGM; Abbott, Marne-la-Vallée, France). The system used in this study measured for up to 14 days every 15 min the glucose profiles in the interstitial fluid using a sensor and a reader. The CGM sensor was placed on the upper back of the arm preferred by the participant and following the manufacturer instructions. If necessary, the sensors were additionally secured by covering them with medical tape (leaving a hole in the middle on top of the sensor for moisture removal). When a sensor was lost before the end of the 14-day period, it was replaced with a new one. Every participant received an accompanying reader and was instructed to read out the sensor every eight hours. The screen of the reader was covered to ensure that participants were blinded to their own glucose read-outs. Each participant wore a CGM twice; the first covering baseline and the run-in period and second during the first 14 days of the intervention period. At the end of each period data was transferred from the reader to a computer using the available Freestyle Libre App.

CGM readouts were analysed by an in-house developed open-source R script, CGM Shiny (Hangelbroek, 2021). CGM metrics were calculated for periods of three consecutive

days covering the same weekdays (Tuesday, Wednesday, Thursday) in the baseline, the run-in and the intervention period. We omitted the first hours until midnight from analysis, also when sensors were replaced due to sensor loss. The first day of sensor readings was omitted from analysis as recommended (Bailey et al., 2015). Calculations of CGM metrics started at midnight (12 am). CGM Shiny can calculate metrics using either data from all 24 h of a day (starting at midnight), or from the period subjects are awake (6:00 am to 12:00 am) or asleep (12:00 am to 6:00 am) as recommended (Danne et al., 2017). Despite precise instructions, it proved difficult for participants to scan the sensor every 8 h to transfer data to the reader. Data gaps of less than an hour (<4 datapoints) were linearly interpolated. Larger gaps were left as missing data. One subject was excluded from data analysis due to sensor failure. From the CGM metrics available we selected the coefficient of variation as a measure of glucose variability expressed as percentage, since it is generally well understood (Danne et al., 2017) and as we observed that it strongly correlated with other glucose variability CGM metrics. To understand the effect of missing data on the outcomes of the CV we conducted a sensitivity analysis. We assessed the percentages of missing data and determined a threshold at 20% missing data. Consequently, the subjects that had more than 20% missing data were excluded from the analysis (n=4 for the placebo and n=6 for the treatment group). Furthermore, we identified and extreme outlier in the placebo group (more than three times interquartile range above third quartile or below first quartile), which appeared to substantially influence the result and, hence, was excluded during the sensitivity analysis. Similarly, an outlier was excluded from analysis when investigating differences in CV after *Blautia* baseline segmentation. Following data exclusion, baseline CV was 21.3% for treatment versus 19.7% for placebo with (p = 0.25).

1.9 Statistical analysis

The study samples size was based on the detection of a mean within-individuals reduction in fasting insulin levels of 29 pmol/L with a two-sided 5% significance level and a power of 80% requiring 27 subjects per study arm. This sample size is sufficient to detect a mean decrease of 29 pmol/L, which is lower than the 34 pmol/L found earlier with ITF (Guess et al., 2016). To account for a potential dropout rate of 10%, a final sample size of 60 patients (30 per study arm) was used. Normality of the outcome variables was assessed by inspecting Q-Q-plots. Depending on normality corresponding parametric or nonparametric testing was applied. For baseline characteristics data was expressed as mean and SD or median and IQR depending on normality to indicate spread in the study population. Changes in biochemical markers, anthropometric measures, and bowel function were expressed as absolute change and as percentage change (relative change). Statistical inference was performed on absolute changes only. The R package mare (Korpela, 2016) was used for gut microbiota analysis uses tools from the R packages vegan (Oksanen et al., 2019), nlme (Pinheiro et al., 2020), MASS (Venables & Ripley, 2002) and glmmADMB (Fournier et al., 2012; Skaug et al., 2016). Multivariate analysis for microbial composition was done using implemented Principle Coordinates Analysis with Bray-Curtis dissimilarities (Korpela, 2016). Changes in levels of individual taxa were assessed by implemented repeated measures analysis, for which the mare package uses subjects as random factor and also automatically performs false-discovery rate correction. The package checks model assumptions and consecutively fits alternative models, which in case of assumption violation by all fitted models leads to the production of no *P*-values and estimates (Korpela, 2016). To assesses differences in changes in levels of faecal SCFA over the intervention periods between the treatment groups we used using linear mixed models, as implemented in the lme4 R package (Bates et al., 2015) that

uses by default an unstructured variance-covariance matrix. We included SCFA as dependent variable and as fixed effect intervention group and intervention period as well as their interaction term and as random effect the subject. Statistical significance levels were calculated using the lmerTest R package (Kuznetsova et al., 2017), which estimated degrees of freedom and *P*-values based on the Satterthwaite's method. Changes over time in stool softness and glycaemic variability were analysed by repeated measures mixed ANOVA (RM ANOVA) with intervention group as between-subject factor, intervention period as within-subject factor, intervention × study period as interaction term and pairwise t-test as post-hoc test with FDR correction. Changes in stool frequency were assessed by Friedman's test with Wilcoxon signed rank-test as post-hoc test with FDR correction. RM ANOVA and Friedman test were performed using the rstatix R package (Kassambara, 2021). Static glycaemic markers and circulating SCFA levels were assessed for differences after the study period between treatment groups by ANCOVA (using baseline as covariate) and for changes over baseline and comparison between groups by paired or unpaired t-test to explore the modulatory potential of the treatment on biomarkers.

1.10 Responder and further subgroup analysis

To further explore gut microbiota differences in relation to metabolic responses, we divided the treatment group based on the relative change in HOMA-ir into responders and non-responders. Subjects with a reduction in HOMA-ir larger than 10% were defined as responders (n = 8), subjects with an increase in HOMA-ir larger than 10% were defined as non-responders (n = 12). Subjects with relatively unchanged HOMA-ir (eight subjects) were excluded (Supplemental Figure S1). Analysis of differences in baseline microbiota highlighted *Blautia* spp. as major differentiating factor between responders and non-responders

($p = 0.01$). This finding was also sustained ($p = 0.02$) when we set the responder definition threshold less stringent to 0% (responder = more than 0% reduction and n = 10; non-responder = no reduction or more than 10% increase and n = 18). The *Blautia* spp. median relative abundance was subsequently used to divide the treatment group into high (n = 14) and low (n = 14) baseline abundance groups. This division was further used to analyse changes in static and dynamic glycaemic biomarkers and fasting circulating SCFA in the treatment group.

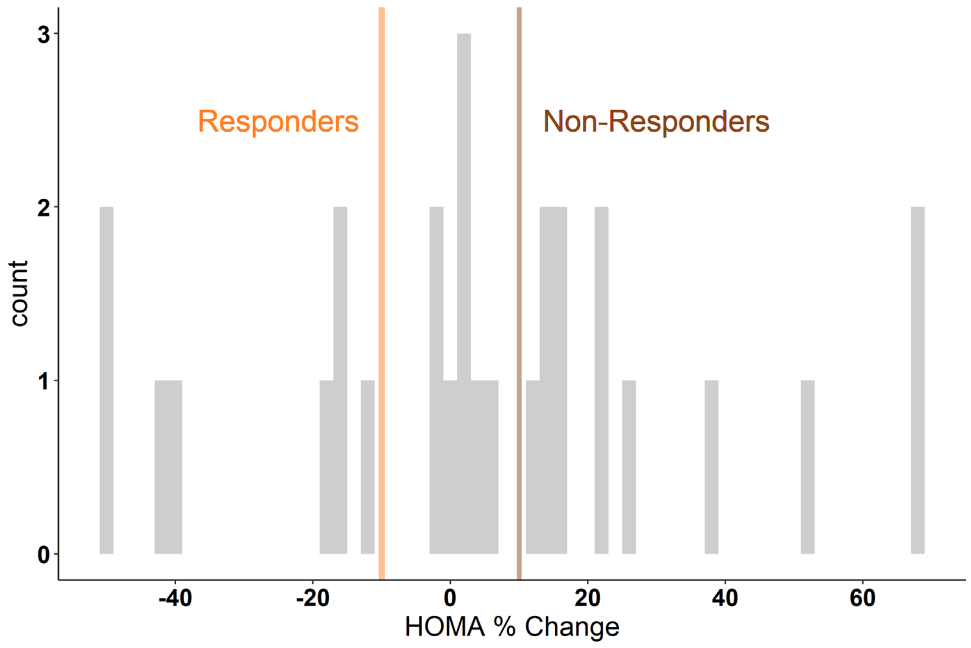

Figure S1 Division of HOMA-responder and non-responders of the treatment group using a 10% cut-off (vertical lines) and excluding subjects with relatively unchanged HOMA-ir (n=8).

SUPPLEMENTARY RESULTS

Participants baseline information

In the Placebo group one participant dropped out during the run-in period due to chest pain, another was excluded from analysis due to missing samples and a third due to medically advised lifestyle changes during the intervention period. In the treatment group one participant dropped out during the run-in period due to an inflammation of the inner ear, another was excluded from analysis due to early discontinuation of the treatment (protocol violation). One participant in the treatment group developed an eczema one week into the study. As the symptoms worsened after the end of the intervention and no earlier allergic reactions to chicory were reported, a possible causal relation with the treatment was excluded.

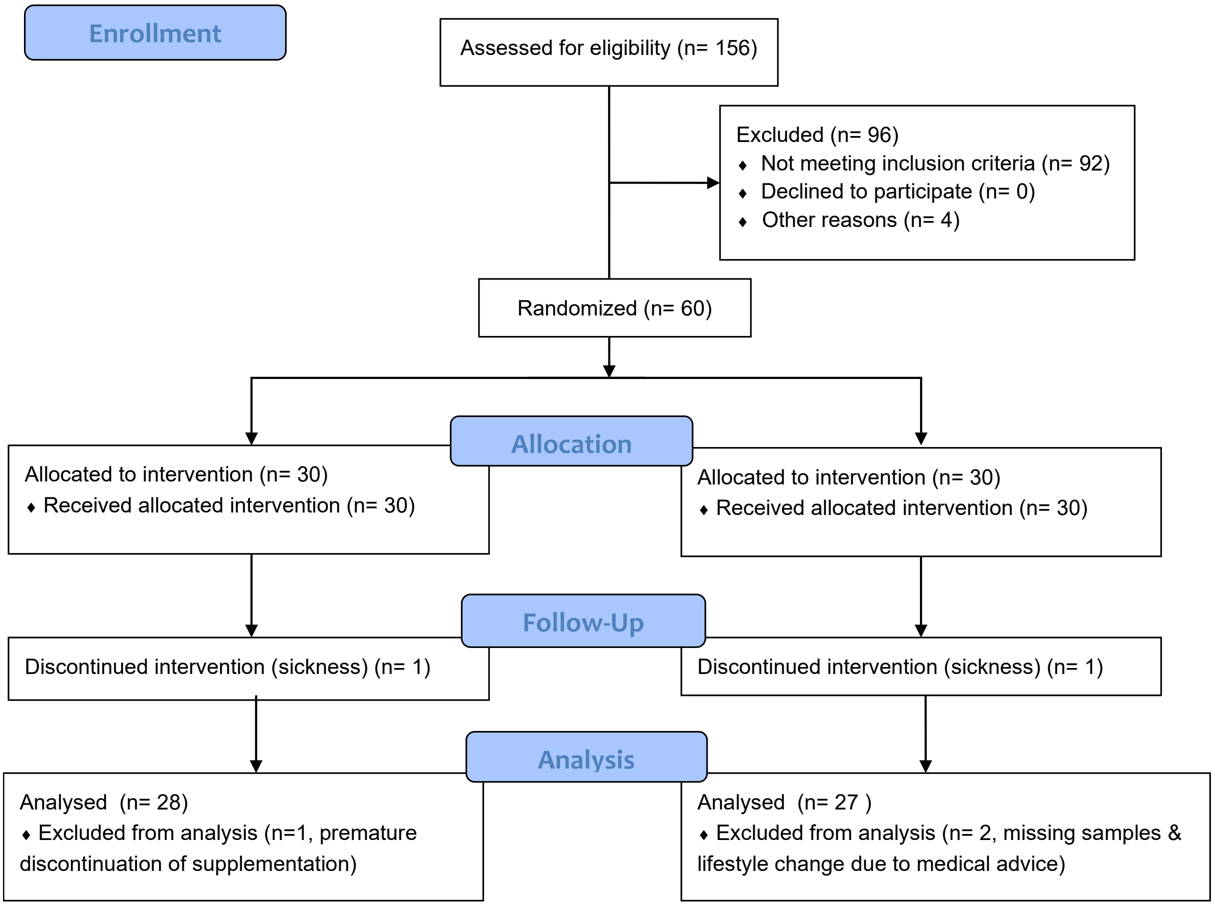

Figure S2 Consort Statement Flow Diagram

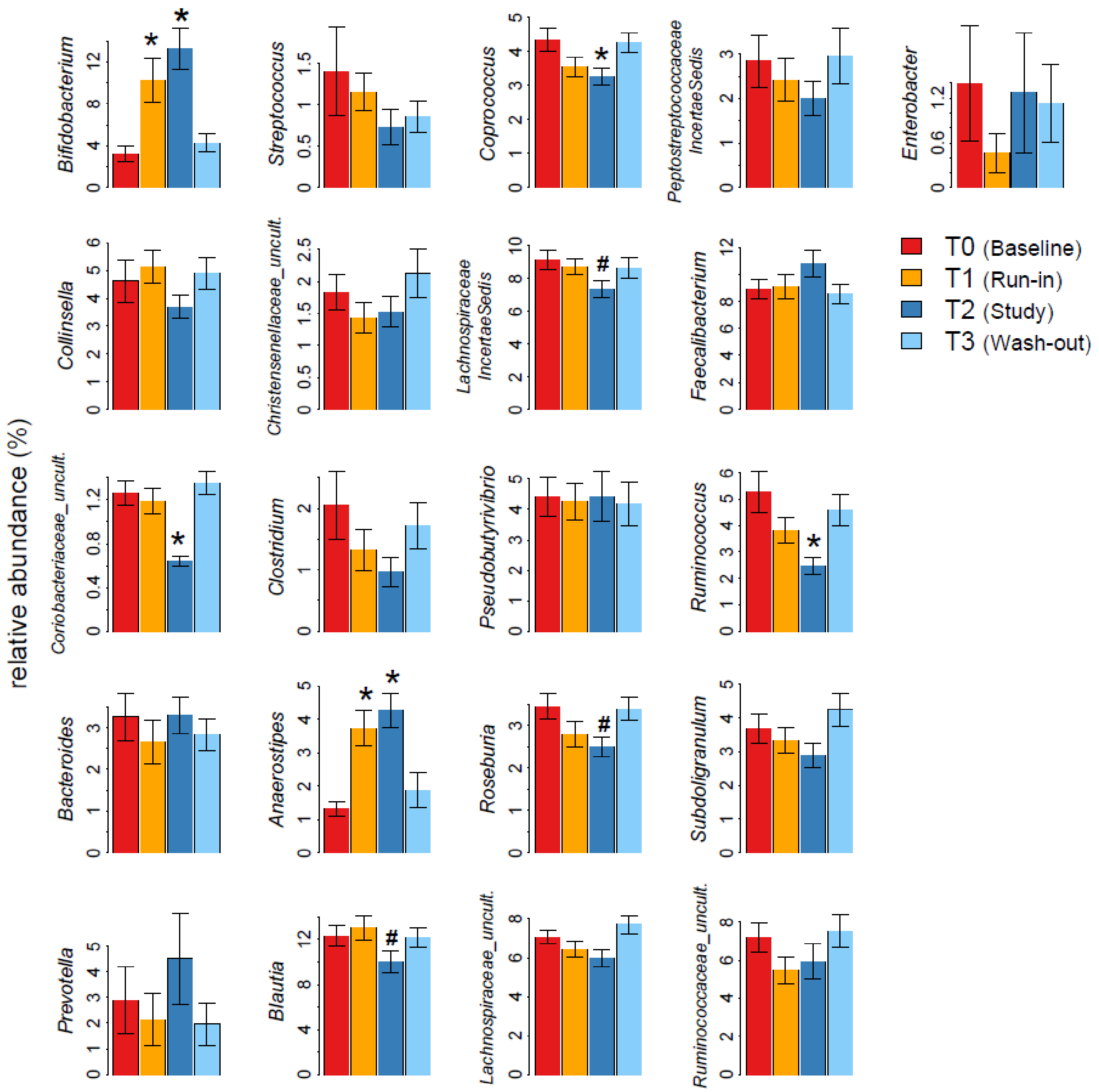

Figure S3 Mean relative abundances of abundant taxa (i.e. mean relative abundance of at least 1% in the whole dataset) in the treatment group at Baseline (T0), after two weeks of 15 g/day treatment intake (T1), after three weeks of 30 g/day treatment intake (T2) and after two weeks washout (T3). **** q<0.001, # q < 0.01 for differences from baseline (T0)***

Table S2 Changes in mean relative abundances of abundant taxa[1] in the treatment group from Baseline (T0) to 15 g/day treatment intake for two weeks (T1) to 30 g/day treatment intake for three weeks (T2) and differences in those changes from the placebo group.[2]

taxon	T0	T1	T2	ΔT1	ΔT2	Fold ΔT1	Fold ΔT2	T1-T0		T2-T0		ΔT1 vs Placebo		ΔT2 vs Placebo	
								p-value	q-value	p-value	q-value	p-value	q-value	p-value	q-value
Bifidobacterium	3.2%	10.1%	13.1%	6.9%	9.9%	3.17	4.09	**<0.001**	**<0.001**	**<0.001**	**<0.001**	0.006	0.050	**<0.001**	**<0.001**
Collinsella	4.4%	5.1%	3.7%	0.7%	-0.7%	1.16	0.83	0.069	0.154	0.365	0.425	0.789	0.876	0.450	0.495
Coriobacteriaceae uncultured	1.2%	1.2%	0.6%	-0.1%	-0.6%	0.93	0.51	0.400	0.514	**<0.001**	**<0.001**	0.825	0.876	-	-
Bacteroides	3.2%	2.7%	3.3%	-0.5%	0.1%	0.83	1.02	-	-	-	-	-	-	0.270	0.393
Prevotella	2.7%	2.1%	3.8%	-0.7%	1.1%	0.76	1.41	0.756	0.756	0.008	0.015	-	-	-	-
Streptococcus	1.1%	1.2%	0.7%	0.1%	-0.3%	1.08	0.68	0.343	0.514	0.377	0.425	0.575	0.876	-	-
Christensenellaceae uncultured	1.8%	1.4%	1.5%	-0.4%	-0.3%	0.79	0.84	0.046	0.118	0.104	0.133	0.941	0.941	0.464	0.495
Clostridium	1.8%	1.3%	1.0%	-0.5%	-0.9%	0.71	0.53	0.170	0.277	0.017	0.026	0.461	0.872	-	-
Anaerostipes	1.3%	3.7%	4.3%	2.4%	3.0%	2.82	3.24	**<0.001**	**<0.001**	**<0.001**	**<0.001**	**<0.001**	**<0.001**	**<0.001**	**<0.001**
Blautia	12.3%	13.0%	10.0%	0.7%	-2.3%	1.06	0.81	0.547	0.616	**0.003**	**0.007**	0.573	0.876	0.682	0.682
Coprococcus	4.3%	3.5%	3.3%	-0.8%	-1.0%	0.82	0.76	0.004	0.023	**<0.001**	**<0.001**	0.053	0.226	0.207	0.332
Lachnospiraceae IncertaeSedis	9.1%	8.7%	7.4%	-0.4%	-1.8%	0.95	0.81	0.459	0.550	**<0.001**	**0.001**	0.664	0.876	0.176	0.332
Pseudobutyrivibrio	4.4%	4.3%	4.4%	-0.2%	0.0%	0.96	1.00	0.666	0.705	0.420	0.444	-	-	0.187	0.332
Roseburia	3.4%	2.7%	2.5%	-0.7%	-0.9%	0.80	0.72	0.012	0.043	**0.001**	**0.002**	0.447	0.872	0.340	0.453
Lachnospiraceae uncultured	7.0%	6.4%	6.0%	-0.6%	-1.1%	0.91	0.85	0.141	0.254	0.016	0.026	0.028	0.157	0.140	0.332
Peptostreptococcaceae IncertaeSedis	2.8%	2.4%	2.0%	-0.4%	-0.8%	0.86	0.71	-	-	-	-	0.680	0.876	-	-
Faecalibacterium	8.9%	9.1%	10.8%	0.2%	1.9%	1.02	1.21	-	-	-	-	0.788	0.876	0.463	0.495
Ruminococcus	5.1%	3.8%	2.5%	-1.3%	-2.6%	0.75	0.48	0.027	0.082	**<0.001**	**<0.001**	0.176	0.500	**<0.001**	**<0.001**
Subdoligranulum	3.7%	3.3%	2.8%	-0.4%	-0.8%	0.89	0.78	0.386	0.514	0.020	0.027	0.308	0.749	0.149	0.332
Ruminococcaceae uncultured	7.2%	5.4%	5.9%	-1.7%	-1.3%	0.76	0.81	0.009	0.042	0.012	0.021	0.131	0.445	0.181	0.332
Enterobacter	1.1%	0.5%	0.9%	-0.6%	-0.2%	0.44	0.85	0.141	0.254	0.465	0.465	-	-	0.165	0.332

[1] Abundant taxa represent those with a mean relative abundance of at least 1% in the whole dataset.

[2] Fold ΔT1 = Fold change after 15 g/day treatment; Fold ΔT2 change after 30 g/day of treatment; T1-T0 = comparison between 15 g/day treatment (T1) and baseline (T0) within the treatment group; T2-T0 = comparison between 30 g/day treatment (T2) and baseline (T0) within the treatment group; ΔT1 vs Placebo = comparison of change over baseline after 15 g/day treatment between the treatment group and the placebo group; ΔT2 vs Placebo = comparison of change over baseline after 30 g/day treatment between the treatment group and the placebo group, - represents not calculable estimates and p- and q-values (Korpela, 2016))

Table S3 Mean relative abundances of abundant taxa[1] in the treatment versus placebo group and differences in these taxa between groups at Baseline (T0), after 15 g/day treatment or 8 g/day iso-caloric placebo intake for two weeks (1), after 30 g/day treatment or 16 g/day iso-caloric placebo intake for three weeks (T2) and after washout of two weeks (T3).[2]

	**T0**					**T1**					**T2**					**T3**				
**taxon**	**P**	**T**	**FoldΔ$_{T-P}$**	**p-value**	**q-value**	**P**	**T**	**FoldΔ$_{T-P}$**	**p-value**	**q-value**	**P**	**T**	**FoldΔ$_{T-P}$**	**p-value**	**q-value**	**P**	**T**	**FoldΔ$_{T-P}$**	**p-value**	**q-value**
Bifidobacterium	4.8%	3.1%	0.65	0.190	0.792	5.5%	10.0%	1.82	0.059	0.615	4.5%	13.0%	2.88	**<0.001**	**0.001**	5.4%	4.3%	0.79	0.447	0.948
Collinsella	4.1%	4.5%	1.09	0.729	0.942	4.6%	5.1%	1.11	0.696	0.858	3.2%	3.7%	1.14	0.468	0.655	4.6%	4.9%	1.07	0.722	0.948
Coriobacteriaceae uncultured	1.5%	1.3%	0.86	0.282	0.821	1.5%	1.2%	0.81	0.126	0.660	1.4%	0.6%	0.47	**<0.001**	**<0.001**	1.7%	1.4%	0.80	0.123	0.835
Bacteroides	3.4%	3.3%	0.96	0.942	0.942	2.1%	2.6%	1.25	0.646	0.858	4.2%	3.3%	0.77	0.879	0.885	3.1%	2.8%	0.92	0.690	0.948
Prevotella	3.4%	2.6%	0.77	0.647	0.942	3.4%	1.9%	0.58	0.277	0.836	4.3%	4.2%	0.97	0.614	0.806	2.2%	1.8%	0.83	0.701	0.948
Streptococcus	1.2%	1.2%	0.96	0.313	0.821	1.3%	1.2%	0.91	0.748	0.858	1.7%	0.7%	0.44	0.015	0.062	1.3%	0.9%	0.67	0.234	0.835
Christensenellaceae uncultured	2.0%	1.8%	0.92	0.693	0.942	1.5%	1.4%	0.95	0.824	0.865	1.8%	1.5%	0.83	0.421	0.655	1.8%	2.1%	1.18	0.651	0.948
Clostridium	1.8%	1.9%	1.08	0.842	0.942	1.6%	1.3%	0.82	0.562	0.858	2.1%	1.0%	0.47	0.075	0.261	1.4%	1.7%	1.23	0.526	0.948
Anaerostipes	1.3%	1.3%	1.02	0.932	0.942	1.6%	3.7%	2.23	**<0.001**	**<0.001**	1.4%	4.3%	3.08	**<0.001**	**<0.001**	1.8%	1.7%	0.91	0.704	0.948
Blautia	12.1%	12.3%	1.02	0.865	0.942	13.7%	13.0%	0.95	0.660	0.858	10.4%	9.9%	0.96	0.726	0.847	11.8%	12.1%	1.02	0.824	0.948
Coprococcus	3.4%	4.3%	1.27	0.035	0.728	3.8%	3.5%	0.93	0.506	0.858	3.3%	3.3%	0.98	0.885	0.885	3.7%	4.3%	1.15	0.170	0.835
IncertaeSedis	7.7%	9.1%	1.19	0.073	0.769	8.3%	8.7%	1.05	0.531	0.858	7.5%	7.4%	0.98	0.840	0.885	8.8%	8.6%	0.98	0.883	0.948
Pseudobutyrivibrio	3.6%	4.4%	1.23	0.426	0.942	4.2%	4.3%	1.02	0.949	0.949	2.7%	4.4%	1.63	0.140	0.327	3.0%	4.2%	1.38	0.278	0.835
Roseburia	3.6%	3.4%	0.96	0.805	0.942	3.1%	2.8%	0.88	0.347	0.836	2.8%	2.5%	0.88	0.363	0.635	2.9%	3.4%	1.18	0.261	0.835
Lachnospiraceae uncultured	6.4%	7.0%	1.10	0.226	0.792	7.2%	6.4%	0.90	0.216	0.836	6.6%	6.0%	0.91	0.345	0.635	6.9%	7.7%	1.11	0.259	0.835
Peptostreptococcaceae IncertaeSedis	3.2%	2.8%	0.88	0.689	0.942	2.9%	2.4%	0.83	0.594	0.858	3.4%	2.0%	0.59	0.109	0.285	3.1%	3.0%	0.95	0.903	0.948
Faecalibacterium	8.6%	8.9%	1.04	0.845	0.942	8.7%	9.1%	1.05	0.777	0.858	9.7%	10.8%	1.12	0.441	0.655	8.9%	8.5%	0.96	0.798	0.948
Ruminococcus	5.0%	5.2%	1.03	0.881	0.942	4.6%	3.8%	0.83	0.358	0.836	5.0%	2.5%	0.50	**<0.001**	**0.002**	5.1%	4.6%	0.90	0.627	0.948
Subdoligranulum	3.5%	3.7%	1.03	0.832	0.942	3.7%	3.3%	0.89	0.494	0.858	3.5%	2.9%	0.82	0.292	0.612	3.4%	4.2%	1.24	0.256	0.835
Ruminococcaceae uncultured	8.7%	7.2%	0.83	0.150	0.785	7.0%	5.4%	0.77	0.097	0.660	7.9%	5.9%	0.75	0.098	0.285	7.5%	7.5%	1.00	0.977	0.977
Enterobacter	0.4%	1.0%	2.80	0.118	0.785	0.9%	0.5%	0.52	0.325	0.836	1.3%	1.0%	0.76	0.691	0.847	0.9%	1.0%	1.10	0.888	0.948

[1] Abundant taxa represent those with a mean relative abundance of at least 1% in the whole dataset.

[2] T=treatment, P=placebo, Fold Δ$_{T-P}$=fold difference in mean relative abundances between treatment and placebo, - represents not calculable estimates and p- and q-values (Korpela, 2016))

Table S4 Changes in abundant taxa[1] in the placebo group from baseline (T0) to 8 g/day placebo intake for two weeks (T1) to 16 g/day placebo intake for three weeks (T2). Intake of 8 g/day placebo and 16 g/day placebo product are iso-calorically corresponding amounts of 15 g/day and 30 g/day treatment product, respectively.[2]

taxon	T0	T1	T2	ΔT1	Δ T2	FoldΔ T1	FoldΔ T2	T1-T0		T2-T0	
								p-value	q-value	p-value	q-value
Bifidobacterium	4.8%	5.3%	4.5%	0.5%	-0.3%	1.11	0.95	0.646	0.794	0.355	0.632
Collinsella	4.0%	4.6%	3.2%	0.6%	-0.8%	1.15	0.81	-	-	-	-
Coriobacteriaceae uncultured	1.5%	1.5%	1.4%	0.0%	-0.1%	1.00	0.96	0.827	0.917	0.290	0.632
Bacteroides	3.4%	2.3%	4.2%	-1.1%	0.8%	0.67	1.25	0.041	0.218	0.106	0.565
Prevotella	3.4%	3.4%	4.3%	0.0%	0.9%	0.99	1.26	0.599	0.794	0.335	0.632
Streptococcus	1.2%	1.3%	1.6%	0.1%	0.4%	1.08	1.36	0.917	0.917	0.711	0.812
Christensenellaceae uncultured	2.0%	1.6%	1.9%	-0.5%	-0.2%	0.77	0.92	0.058	0.232	0.552	0.747
Clostridium	1.8%	1.6%	2.1%	-0.2%	0.3%	0.90	1.16	0.570	0.794	0.315	0.632
Anaerostipes	1.3%	1.6%	1.3%	0.4%	0.0%	1.30	1.02	-	-	-	-
Blautia	12.1%	13.7%	10.4%	1.6%	-1.7%	1.13	0.86	0.137	0.365	0.079	0.565
Coprococcus	3.4%	3.8%	3.3%	0.4%	-0.1%	1.12	0.98	0.097	0.312	0.859	0.916
Lachnospiraceae IncertaeSedis	7.7%	8.3%	7.5%	0.6%	-0.2%	1.08	0.98	-	-	-	-
Pseudobutyrivibrio	3.6%	4.2%	2.7%	0.6%	-0.9%	1.18	0.76	0.867	0.917	0.254	0.632
Roseburia	3.6%	3.1%	2.9%	-0.4%	-0.7%	0.88	0.81	0.536	0.794	0.105	0.565
Lachnospiraceae uncultured	6.4%	7.2%	6.6%	0.8%	0.2%	1.12	1.03	0.014	0.114	0.581	0.747
Peptostreptococcaceae IncertaeSedis	3.2%	2.9%	3.4%	-0.3%	0.2%	0.91	1.06	0.334	0.679	0.974	0.974
Faecalibacterium	8.6%	8.7%	9.7%	0.1%	1.1%	1.01	1.12	0.487	0.794	0.607	0.747
Ruminococcus	5.0%	4.6%	5.0%	-0.4%	0.0%	0.91	1.00	0.340	0.679	0.591	0.747
Subdoligranulum	3.5%	3.7%	3.5%	0.1%	-0.1%	1.03	0.98	-	-	-	-
Ruminococcaceae uncultured	8.7%	7.0%	7.8%	-1.6%	-0.8%	0.81	0.91	0.009	0.114	0.207	0.632
Enterobacter	0.4%	0.9%	1.1%	0.6%	0.7%	2.63	2.95	-	-	-	-

[1] Abundant taxa represent those with a mean relative abundance of at least 1% in the whole dataset.

[2] Fold ΔT1 = Fold change after 8 g/day placebo intake; Fold ΔT2 change after 16 g/day placebo; T1-T0 = comparison between 8 g/day placebo (T1) and baseline (T0) within the placebo group; T2-T0 = comparison between 16 g/day placebo (T2) and baseline (T0) within the placebo group; - represents not calculable estimates and p- and q-values (Korpela, 2016))

Fold-changes in the treatment group subjects with low or high baseline *Bifidobacterium* spp. and *Anaerostipes* spp. relative abundances.

Table S5 Differences in fold-change between subjects of the treatment group with high or low baseline relative abundance (5) of *Bifidobacterium* and *Anaerostipes* spp. based on baseline median division.[1]

**Group**		**Relative abundance (%) T0**	**Relative abundance (%) T2**	**Fold-change T2**
Bifidobacterium spp.	High (n=14)	5.67 ± 1.21	18.67 ± 2.98	4.23
	Low (n=14)	0.72 ± 0.18	7.84 ± 1.72	11.55
Anaerostipes spp	High (n=14)	2.04 ± 0.32	5.31 ± 0.78	2.74
	Low (n=14)	0.60 ± 0.05	3.24 ± 0.55	6.12

[1] Data is presented as mean with SEM in parentheses. T0 = baseline; T2 = 30 g/day treatment; Fold-change T2 = fold change after 30 g/day treatment over baseline.

Table S6 Estimated model fixed effects of Linear Mixed Model analysis of changes in faecal short-chain fatty acids (SCFA) between the intervention groups (treatment versus placebo) over the intervention periods from baseline to run-in (15 g/day treatment or 8 g/day iso-caloric placebo), study (30 g/day treatment or 16 g/day iso-caloric placebo) and washout (no product intake).

Effect		Estimate	Std. error	df	t-value	p-value
Total faecal SCFA (mmol/kg)						
Intercept		56.50	5.66	135.28	9.98	<2e-16
Intervention group	treatment	-5.86	7.94	135.28	-0.738	0.462
	placebo	0	-	-	-	-
Period	run-in	2.57	6.14	153.20	0.419	0.676
	study	-6.51	6.07	152.65	-1.073	0.285
	washout	-4.87	6.14	153.20	-0.793	0.429
	baseline	0	-	-	-	-
*Intervention*Period*	treatment*run-in	6.99	8.55	152.93	0.818	0.415
	treatment*study	19.53	8.50	152.65	2.297	0.023
	treatment*washout	3.532	8.83	154.36	0.4	0.69
Faecal acetate (mmol/kg)						
Intercept		37.49	3.87	140.59	9.689	<2e-16
Intervention group	treatment	-4.82	5.42	140.59	-0.888	0.3759
	placebo	0	-	-	-	-
Period	run-in	2.04	4.28	153.24	0.477	0.634
	study	-4.44	4.23	152.66	-1.049	0.2959
	washout	-3.57	4.28	153.24	-0.834	0.4058
	baseline	0	-	-	-	-
*Intervention*Period*	treatment*run-in	3.63	5.97	152.96	0.608	0.5443
	treatment*study	13.69	5.93	152.66	2.309	0.0223
	treatment*washout	2.91	6.16	154.47	0.472	0.6374
Faecal propionate (mmol/kg)						
Intercept		9.28	0.96	117.67	9.661	<2e-16
Intervention group	treatment	-0.21	1.35	117.67	-0.157	0.8751
	placebo	0	-	-	-	-
Period	run-in	0.80	0.96	153.01	0.833	0.4063
	study	-0.45	0.95	152.58	-0.476	0.6351
	washout	-0.11	0.96	153.01	-0.113	0.9099
	baseline	0	-	-	-	-
*Intervention*Period*	treatment*run-in	0.80	1.34	152.80	0.595	0.5527
	treatment*study	2.47	1.33	152.58	1.858	0.0651
	treatment*washout	-0.08	1.38	153.93	-0.056	0.9553
Faecal butyrate (mmol/kg)						
Intercept		9.74	1.17	128.61	8.297	1.25e-13
Intervention group	treatment	-0.83	1.65	128.61	-0.502	0.6163
	placebo	0	-	-	-	-
Period	run-in	-0.27	1.24	152.71	-0.217	0.8286
	study	-1.62	1.23	152.20	-1.321	0.1886
	washout	-1.18	1.24	152.71	-0.95	0.3438
	baseline					
*Intervention*Period*	treatment*run-in	2.56	1.73	152.47	1.484	0.1398
	treatment*study	3.36	1.72	152.20	1.959	0.0519
	treatment*washout	0.65	1.78	153.79	0.364	0.716

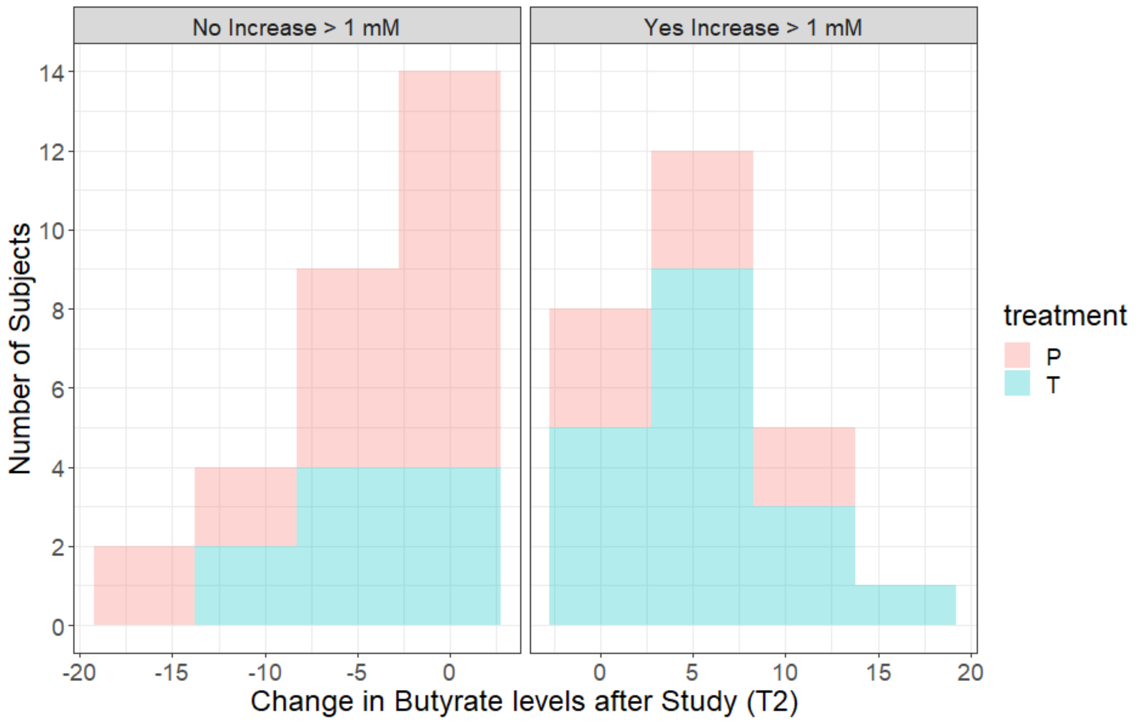

Figure S4 Differences between treatment (T) and placebo (P) in the proportion of subjects increasing in butyrate after three weeks of 30 g/day treatment or iso-caloric placebo intake (T2); with >1 mM being the minimal increase observed in the treatment group. In the treatment 64.3% (n = 18) increased, while 35.7% (n = 10) showed no increase versus 29.6% (n = 8) increase in the placebo and 70.3% (n = 19) showing no increase (Fisher's exact test p = 0.015).

Post-intervention differences at only T2 between groups

Table S7 Post-intervention differences corrected for baseline between the treatment (T) and the placebo (P) after the intervention period (T2) of three weeks intake of 30g/day treatment or 16 g/day iso-caloric placebo product. Adjusted means and mean differences assessed by analysis of covariance (ANCOVA with baseline as covariate). No ANCOVA was done for bowel function measurements due to model violation. [1]

		Treatment	Placebo		
	**adjusted mean T0**	**Adjusted mean T2**	**adjusted mean T2**	**Adjusted mean difference T2 T vs P**	***p-value adjusted mean difference T2 T vs P***
**Faecal SCFA**					
Total SCFA (mmol/kg)	53.50	64.60 (5.04)	49.10 (5.14)	+ 15.50 (7.22)	***0.036***
Acetate (mmol/kg)	35.00	42.50 (3.44)	32.40 (3.50)	+ 10.10 (4.93)	***0.045***
Propionate (mmol/kg)	9.17	11.10 (0.82)	8.77 (0.84)	+2.38 (1.17)	***0.048***
Butyrate (mmol/kg)	9.32	10.80 (0.99)	7.94 (1.01)	+ 2.89 (1.42)	***0.047***
**Circulating fasting SCFA**					
Total SCFA (µM)	50.50	57.40 (3.48)	48.90 (3.54)	+ 8.56 (4.97)	*0.091*
Acetate (µM)	44.90	51.60 (3.39)	44.10 (3.46)	+ 7.47 (4.85)	*0.129*
Propionate (µM)	5.31	5.61 (0.53)	4.76 (0.54)	+ 0.85 (0.76)	*0.266*
Butyrate (µM)	0.25	0.15 (0.06)	0.08 (0.6)	+ 0.06 (0.08)	*0.451*
**Glucose Homeostasis**					
HOMA-ir	1.31	1.26 (0.07)	1.31 (0.07)	-0.05 (0.09)	*0.570*
Fasting insulin (µU/mL)	8.31	8.05 (0.42)	8.36 (0.43)	-0.32 (0.60)	*0.597*
Fasting Glucose (mmol/L)	6.01	5.92 (0.08)	5.93 (0.08)	-0.02 (0.12)	*0.877*

[1] Data is presented as mean with SEM in parentheses

Changes in static glycaemic markers

Table S8 Changes in HOMA-ir and fasting glucose and insulin levels after the intervention period consisting of three weeks intake of 30g/day treatment or 16 g/day iso-caloric placebo product. [1]

	Treatment (n=28)				Placebo (n=27)				*p-value* $\Delta_T - \Delta_P$
	T0	T2	Δ_T	*p-value*	T0	T2	Δ_P	*p-value*	
**HOMA-ir†**	1.28 ± 0.12	1.24 ± 0.09	-0.04 ± 0.08	0.566	1.34 ± 0.12	1.34 ± 0.12	0.00 ± 0.07	0.790	0.877
**Fasting glucose, *mmol/L***	6.11 ± 0.11	5.98 ± 0.11	-0.29 ± 0.04	0.142	5.91 ± 0.11	5.87 ± 0.11	-0.04 ± 0.09	0.637	0.519
**Fasting insulin, *µU/mL***	8.05 ± 0.73	7.87 ± 0.58	-0.19 ± 0.50	0.713	8.57 ± 0.77	8.55 ± 0.77	-0.02 ± 0.43	0.966	0.802

[1] Data is presented as mean with SEM in parentheses.† represents analysed using non-parametric testing

Glycaemic variability assessed by coefficient or variation from continuous glucose monitoring

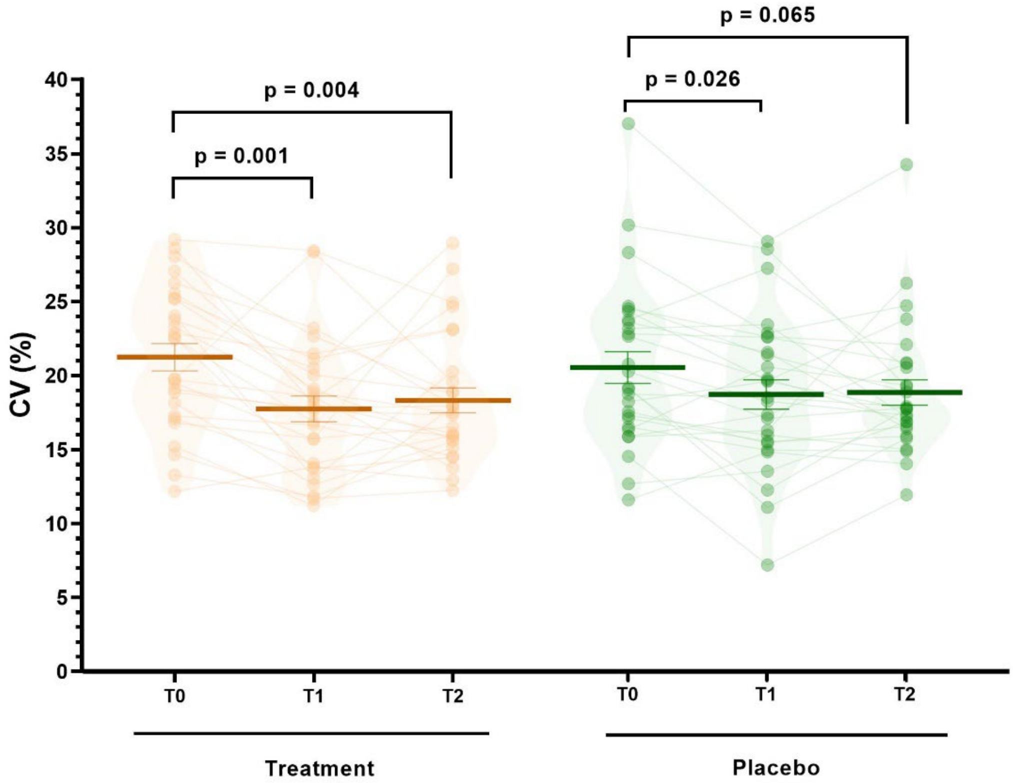

Figure S5 Coefficient of variation (CV%) as a measure of glycaemic control assessed in the treatment group (n=27) and the placebo group (n=27) using continuous glucose measurement on three same consecutive weekdays during baseline (T0), the run-in period (T1) with 15 g/day treatment or 8 g/day iso-caloric placebo, and during the study period (T2) with 30 g/day treatment or 16 g/day isocaloric placebo (repeated measures ANOVA with main effect of period p<0.001, post-hoc tests with FDR-adjustment). No difference between groups in baseline CV was observed before (p = 0.55) and after sensitivity analysis (p = 0.25).

Changes in static glycaemic markers in subject with high or low baseline *Blautia* spp. relative abundance

Table S9 Changes in HOMA-ir and fasting glucose and insulin levels in the treatment group in subjects with either low or high baseline relative abundance of *Blautia* spp. after the intervention period consisting of three weeks of 30 g/day treatment.[1]

	**Low *Blautia* spp. (n=14)**				**High *Blautia* spp. (n=14)**				
	**T0**	**T2**	**Δ_T**	***p-value***	**T0**	**T2**	**Δ_P**	***p-value***	***p-value* $\Delta_L - \Delta_H$**
**HOMA-ir†**	1.30 ± 0.18	1.16 ± 0.15	-0.14 ± 0.10	0.289	1.25 ± 0.15	1.32 ± 0.10	0.07 ± 0.12	0.055	0.045
**Fasting glucose, *mmol/L***	6.06 ± 0.13	5.75 ± 0.11	-0.31 ± 0.11	0.013	6.15 ± 0.17	6.21 ± 0.16	0.06 ± 0.10	0.546	0.019
**Fasting insulin, *µU/mL***	8.23 ± 1.17	7.46 ± 0.99	-0.82 ± 0.65	0.229	7.83 ± 0.91	8.28 ± 0.63	0.45 ± 0.75	0.556	0.210

[1] Data is presented as mean with SEM in parentheses. † represents analysed using non-parametric testing

Fasting circulating short-chain fatty acid levels in subject with high or low baseline *Blautia* spp. relative abundance

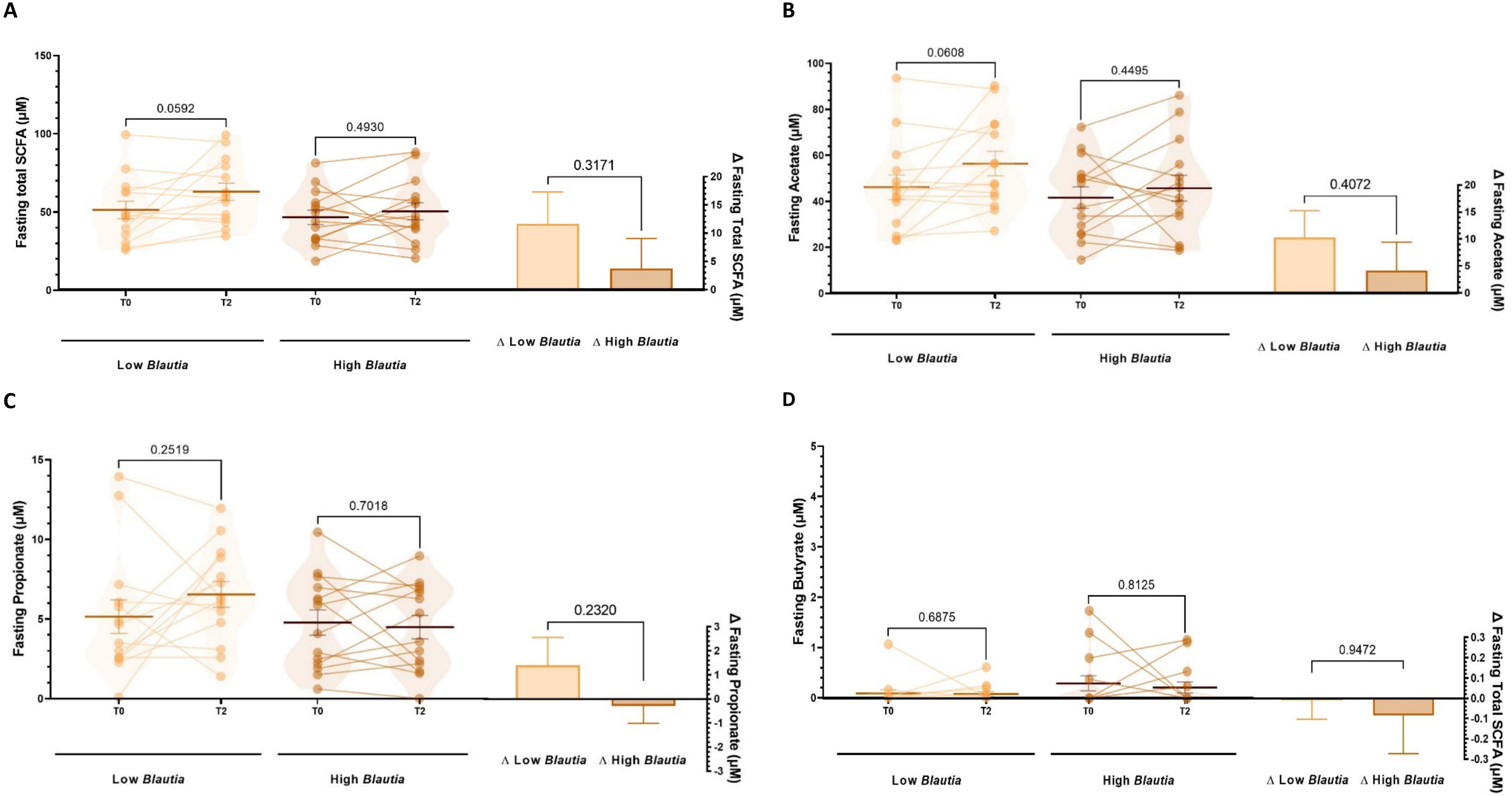

Figure S6 Effect of *Blautia* baseline abundance on changes in fasting circulating short-chain fatty acids (SCFA) after 30 g/day treatment in subjects of the treatment group with either low (n=14) or high (n=14) baseline *Blautia* spp. relative abundance. Butyrate levels were analysed using nonparametric testing. A: fasting circulating total SCFA, B: fasting circulating acetate levels, C: fasting circulating propionate levels, D: fasting circulating butyrate levels.

References

Bailey, T., Bode, B. W., Christiansen, M. P., Klaff, L. J., & Alva, S. (2015). The Performance and Usability of a Factory-Calibrated Flash Glucose Monitoring System. *Diabetes Technology & Therapeutics*, *17*(11), 787–794. https://doi.org/10.1089/dia.2014.0378

Bui, T. P. N., Schols, H. A., Jonathan, M., Stams, A. J. M., de Vos, W. M., & Plugge, C. M. (2019). Mutual Metabolic Interactions in Co-cultures of the Intestinal Anaerostipes rhamnosivorans With an Acetogen, Methanogen, or Pectin-Degrader Affecting Butyrate Production. *Frontiers in Microbiology*, *10*, 2449. https://doi.org/10.3389/fmicb.2019.02449

Camacho, C., Coulouris, G., Avagyan, V., Ma, N., Papadopoulos, J., Bealer, K., & Madden, T. L. (2009). BLAST+: Architecture and applications. *BMC Bioinformatics*, *10*(1), 1–9. https://doi.org/10.1186/1471-2105-10-421/FIGURES/4

Danne, T., Nimri, R., Battelino, T., Bergenstal, R. M., Close, K. L., DeVries, J. H., Garg, S., Heinemann, L., Hirsch, I., Amiel, S. A., Beck, R., Bosi, E., Buckingham, B., Cobelli, C., Dassau, E., Doyle, F. J., Heller, S., Hovorka, R., Jia, W., … Phillip, M. (2017). International Consensus on Use of Continuous Glucose Monitoring. *Diabetes Care*, *40*(12), 1631–1640. https://doi.org/10.2337/DC17-1600

Deleu, S., Machiels, K., Raes, J., Verbeke, K., & Vermeire, S. (2021). Short chain fatty acids and its producing organisms: An overlooked therapy for IBD? *EBioMedicine*, *66*, 103293. https://doi.org/10.1016/J.EBIOM.2021.103293

Edgar, R. C. (2010). Search and clustering orders of magnitude faster than BLAST. *Bioinformatics*, *26*(19), 2460–2461. https://doi.org/10.1093/bioinformatics/btq461

Fournier, D. A., Skaug, H. J., Ancheta, J., Ianelli, J., Magnusson, A., Maunder, M. N., Nielsen, A., & Sibert, J. (2012). AD Model Builder: using automatic differentiation for statistical inference of highly parameterized complex nonlinear models. *Optimization Methods and Software*, *27*(2), 233–249. https://doi.org/10.1080/10556788.2011.597854

Guess, N. D., Dornhorst, A., Oliver, N., & Frost, G. S. (2016). A Randomised Crossover Trial: The Effect

of Inulin on Glucose Homeostasis in Subtypes of Prediabetes. *Annals of Nutrition and Metabolism*, *68*(1), 26–34. https://doi.org/10.1159/000441626

Han, J., Lin, K., Sequeira, C., & Borchers, C. H. (2015). An isotope-labeled chemical derivatization method for the quantitation of short-chain fatty acids in human feces by liquid chromatography-tandem mass spectrometry. *Analytica Chimica Acta*, *854*, 86–94. https://doi.org/10.1016/j.aca.2014.11.015

Hangelbroek, R. (2021). *CGM Shiny. R package version 0.0.0.9*. https://github.com/rolandhangelbroek/cgmshiny/

Kassambara, A. (2021). *rstatix: Pipe-Friendly Framework for Basic Statistical Tests. R package version 0.7.0.* https://cran.r-project.org/package=rstatix

Korpela, K. (2016). *mare: Microbiota Analysis in R Easily. R package version 1.0.* https://doi.org/10.5281/zenodo.50310

Korpela, K., Salonen, A., Hickman, B., Kunz, C., Sprenger, N., Kukkonen, K., Savilahti, E., Kuitunen, M., & de Vos, W. M. (2018). Fucosylated oligosaccharides in mother's milk alleviate the effects of caesarean birth on infant gut microbiota. *Scientific Reports*, *8*(1), 13757. https://doi.org/10.1038/s41598-018-32037-6

Kuznetsova, A., Brockhoff, P. B., & Christensen, R. H. B. (2017). lmerTest Package: Tests in Linear Mixed Effects Models. *Journal of Statistical Software*, *82*(1), 1–26. https://doi.org/10.18637/JSS.V082.I13

Levy, J. C., Matthews, D. R., & Hermans, M. P. (1998). Correct Homeostasis Model Assessment (HOMA) Evaluation Uses the Computer Program. *Diabetes Care*, *21*(12), 2191–2192. https://doi.org/10.2337/diacare.21.12.2191

Matthews, D. R., Hosker, J. P., Rudenski, A. S., Naylor, B. A., Treacher, D. F., & Turner, R. C. (1985). Homeostasis model assessment: insulin resistance and β-cell function from fasting plasma glucose and insulin concentrations in man. *Diabetologia*, *28*(7), 412–419. https://doi.org/10.1007/BF00280883

Oksanen, J., Blanchet, F. G., Friendly, M., Kindt, R., Legendre, P., McGlinn, D., Minchin, P. R., O'Hara, R. B., Simpson, G. L., Solymos, P., Stevens, M. Henry H. Szoecs, E., & Wagner, H. (2019). *vegan: Community Ecology Package. R package version 2.5-6*. https://cran.r-project.org/package=vegan%0A

Pinheiro, J., Bates, D., DebRoy, S., Sarkar, D., & R Core Team. (2020). *nlme: Linear and Nonlinear Mixed Effects Models. R package version 3.1-144*. https://cran.r-project.org/package=nlme

Quast, C., Pruesse, E., Yilmaz, P., Gerken, J., Schweer, T., Yarza, P., Peplies, J., & Glöckner, F. O. (2013). The SILVA ribosomal RNA gene database project: improved data processing and web-based tools. *Nucleic Acids Research*, *41*(Database issue), D590–D596. https://doi.org/10.1093/nar/gks1219

Salonen, A., Nikkilä, J., Jalanka-Tuovinen, J., Immonen, O., Rajilić-Stojanović, M., Kekkonen, R. A., Palva, A., & de Vos, W. M. (2010). Comparative analysis of fecal DNA extraction methods with phylogenetic microarray: Effective recovery of bacterial and archaeal DNA using mechanical cell lysis. *Journal of Microbiological Methods*, *81*(2), 127–134. https://doi.org/10.1016/j.mimet.2010.02.007

Skaug, H., Fournier, D., Bolker, B., Magnusson, A., & Nielsen, A. (2016). *Generalized Linear Mixed Models using "AD Model Builder". R package version 0.8.3.3*. http://glmmadmb.r-forge.r-project.org/

van Dongen, K. C. W., van der Zande, M., Bruyneel, B., Vervoort, J. J. M., Rietjens, I. M. C. M., Belzer, C., & Beekmann, K. (2021). An in vitro model for microbial fructoselysine degradation shows substantial interindividual differences in metabolic capacities of human fecal slurries. *Toxicology in Vitro : An International Journal Published in Association with BIBRA*, *72*, 105078. https://doi.org/10.1016/j.tiv.2021.105078

Venables, W. N., & Ripley, B. D. (2002). *Modern Applied Statistics with S.* (4th ed). Springer. https://www.stats.ox.ac.uk/pub/MASS4/
